# PRC2 targeting is a therapeutic strategy for EZ score defined high-risk multiple myeloma patients and overcome resistance to IMiDs

**DOI:** 10.1186/s13148-018-0554-4

**Published:** 2018-10-03

**Authors:** Laurie Herviou, Alboukadel Kassambara, Stéphanie Boireau, Nicolas Robert, Guilhem Requirand, Carsten Müller-Tidow, Laure Vincent, Anja Seckinger, Hartmut Goldschmidt, Guillaume Cartron, Dirk Hose, Giacomo Cavalli, Jerome Moreaux

**Affiliations:** 1 Department of Biological Hematology, CHU Montpellier, Montpellier, France; 2 IGH, CNRS, Univ Montpellier, Montpellier, France; 3 UFR de Médecine, Univ Montpellier, Montpellier, France; 4 Medizinische Klinik und Poliklinik V, Universitätsklinikum Heidelberg, Heidelberg, Germany; 5 Nationales Centrum für Tumorerkrankungen, Heidelberg, Germany; 6 Department of Clinical Hematology, CHU Montpellier, Montpellier, France; 7 UMR CNRS 5235, Univ Montpellier, Montpellier, France; 8 Laboratory for Monitoring Innovative Therapies, Department of Biological Hematology, Hôpital Saint-Eloi-CHRU de Montpellier, 80, av. Augustin Fliche, 34295 Montpellier, Cedex 5 France

**Keywords:** PRC2, Multiple myeloma, Predictive score, Epigenetics

## Abstract

**Background:**

Multiple myeloma (MM) is a malignant plasma cell disease with a poor survival, characterized by the accumulation of myeloma cells (MMCs) within the bone marrow. Epigenetic modifications in MM are associated not only with cancer development and progression, but also with drug resistance.

**Methods:**

We identified a significant upregulation of the polycomb repressive complex 2 (PRC2) core genes in MM cells in association with proliferation. We used EPZ-6438, a specific small molecule inhibitor of EZH2 methyltransferase activity, to evaluate its effects on MM cells phenotype and gene expression prolile.

**Results:**

PRC2 targeting results in growth inhibition due to cell cycle arrest and apoptosis together with polycomb, DNA methylation, TP53, and RB1 target genes induction. Resistance to EZH2 inhibitor is mediated by DNA methylation of PRC2 target genes. We also demonstrate a synergistic effect of EPZ-6438 and lenalidomide, a conventional drug used for MM treatment, activating B cell transcription factors and tumor suppressor gene expression in concert with MYC repression. We establish a gene expression-based EZ score allowing to identify poor prognosis patients that could benefit from EZH2 inhibitor treatment.

**Conclusions:**

These data suggest that PRC2 targeting in association with IMiDs could have a therapeutic interest in MM patients characterized by high EZ score values, reactivating B cell transcription factors, and tumor suppressor genes.

**Electronic supplementary material:**

The online version of this article (10.1186/s13148-018-0554-4) contains supplementary material, which is available to authorized users.

## Background

Epigenetic events are key mechanisms in the regulation of cell fate and cell identity. DNA methylation, miRNA-associated gene repression, and histone modifications have been implicated in numerous diseases, including cancers and represent new therapeutic targets [1].

Gene expression regulation through polycomb-induced histone modifications is a well-studied mechanism. The polycomb repressive complex 2 (PRC2) contains three core subunits: EED (embryonic ectoderm development), SUZ12 (suppressor of zeste 12 homolog), and EZH2 (enhancer of zeste homolog 2). PRC2 represses gene transcription through tri-methylation of lysine 27 of histone 3 (H3K27me3) by its catalytic subunit EZH2.

EZH2 deregulation has been described in many cancer types, including hematological malignancies. Its overexpression or gain of function mutations lead to abnormal H3K27me3 accumulation, repressing tumor suppressor genes, such as cell cycle inhibitors, apoptotic activators, and senescence and differentiation factors [1].

Multiple myeloma (MM) is a neoplasia characterized by the accumulation of clonal plasma cells within the bone marrow. Recent advances in treatment have led to an overall survival of intensively treated patients of 6–7 years and an event-free survival of 3–4 years [2]. However, patients invariably relapse after multiple lines of treatment, with shortened intervals between relapses, and finally become resistant to all treatments, resulting in loss of clinical control over the disease. MM is a genetically and clinically heterogeneous disease. Genome sequencing studies have revealed considerable heterogeneity and genomic instability, a complex mutational landscape and a branching pattern of clonal evolution [3, 4]. Epigenetic marks such as DNA methylation or histone posttranslational modifications are also involved in MM pathophysiology and drug resistance [5, 6].

Global gene expression profiling indicated that, while *EZH2* is upregulated, its target genes are downregulated in myeloma cells compared with normal plasma cells [7]. In human MM cell lines (HMCL), *EZH2* expression has been correlated with increased proliferation and an independence on growth factors [8]. Inhibition of EZH2 expression and activity is associated with HMCL growth inhibition [9, 10] and decreased tumor load in a mouse model of MM [7, 11]. One study shows that this effect is related to epithelial tumor suppressor gene upregulation [11]. However, the use of specific EZH2 inhibitors demonstrated that MM proliferation inhibition is time dependent and cell line specific, indicating that EZH2 does not play a universal and monotonous role in promoting MM [11]. Furthermore, the first genome-wide profiling of H3K27me3 and H3K4me3 in MM patient samples was recently published, showing a unique epigenetic profile of primary MM cells compared to normal bone marrow plasma cells [10]. EZH2 inhibition was associated with upregulation of microRNAs with potential tumor suppressor functions [12]. More recently, EZH2 overexpression was reported to be associated with poor outcome and dysregulation of proliferation [13]. These data underscore an oncogenic role of EZH2 in MM. EZH2 inhibitors are currently in phase 2 clinical development in relapsed or refractory non-Hodgkin lymphoma (NHL) and biomarkers are needed for patient selection since neither EZH2 mutations nor H3K27me3 levels are sufficient to predict NHL cell response to EZH2 inhibitors [1, 14].

Here, we identified that PRC2 core genes are overexpressed in MM cells in association with proliferation activation. Treatment of MM cells with EPZ-6438, a specific small molecule inhibitor of EZH2 methyltransferase activity, results in growth inhibition due to cell cycle arrest and apoptosis. Resistance to EZH2 inhibitor is mediated by DNA methylation of PRC2 target genes. We also observed a synergy between EPZ-6438 and lenalidomide, a conventional drug used for MM treatment. More interestingly, pretreatment of myeloma cells with EPZ-6438 significantly re-sensitizes drug-resistant MM cells to lenalidomide. EPZ-6438/lenalidomide combination induced a significant transcriptional reprogramming of MM cells targeting major B and plasma cell transcription factors in association with MYC repression. RNA sequencing combined with H3K27me3 ChIP analyses allowed us to build an EZ GEP-based score that is able to predict HMCL and primary MM cell sensitivity to EZH2 inhibitors.

## Methods

### Human myeloma cell lines (HMCLs)

XG human myeloma cell lines were obtained as previously described [15]. JJN3 was kindly provided by Dr. Van Riet (Brussels, Belgium), JIM3 by Dr. MacLennan (Birmingham, UK), and MM1S by Dr. S. Rosen (Chicago, USA). AMO-1, LP1, L363, U266, OPM2, and SKMM2 were purchased from DSMZ (Braunsweig, Germany) and RPMI8226 from ATTC (Rockville, MD, USA). All HMCLs derived in our laboratory were cultured in the presence of recombinant IL-6. HMCLs were authenticated according to their short tandem repeat profiling and their gene expression profiling using Affymetrix U133 plus 2.0 microarrays deposited in the ArrayExpress public database under accession numbers E-TABM-937 and E-TABM-1088 [15].

### Primary multiple myeloma cells

Bone marrow samples were collected after patients’ written informed consent in accordance with the Declaration of Helsinki and institutional research board approval from Heidelberg and Montpellier University Hospital. Bone marrows were collected from 206 patients treated with high-dose Melphalan (HDM) and autologous stem cell transplantation (ASCT), and this cohort is termed “Heidelberg-Montpellier” (HM) cohort [16]. Patients’ MMCs were purified using anti-CD138 MACS microbeads (Miltenyi Biotec, Bergisch Gladbach, Germany) and their gene expression profile (GEP) obtained using Affymetrix U133 plus 2.0 microarrays as described [17]. The CEL files and MAS5 files are available in the ArrayExpress public database (E-MTAB-372). The structural chromosomal aberrations, as well as numerical aberrations, were assayed by fluorescence in situ hybridization (iFISH). We also used publicly available Affymetrix GEP (Gene Expression Omnibus, accession number GSE2658) of a cohort of 345 purified MMC from previously untreated patients from the University of Arkansas for Medical Sciences (UAMS, Little Rock, AR, USA), termed in the following UAMS-TT2 cohort. These patients were treated with total therapy 2 including HDM and ASCT [18]. We also used Affymetrix data from total therapy 3 cohort (UAMS-TT3; *n* = 158; E-TABM-1138) [19] of 188 relapsed MM patients subsequently treated with bortezomib (GSE9782) from the study by Mulligan et al. [20].

### EZH2 inhibition in primary MM cells

BM of patients presenting with previously untreated MM (*n* = 7) at the university hospital of Montpellier was obtained after patients’ written informed consent in accordance with the Declaration of Helsinki and agreement of the Montpellier University Hospital Centre for Biological Resources (DC-2008-417). Mononuclear cells were treated with or without EPZ-6438 (370 nM or 1 μM) and/or lenalidomide (2 μM), and MMC cytotoxicity was evaluated using anti-CD138-phycoerythrin monoclonal antibody (Immunotech, Marseille, France) as described [6].

### Cell growth assay

HMCLs were cultured for 15 days in RPMI 1640 medium, 10% FCS, and 2 ng/ml IL-6 (control medium) in the presence of EPZ-6438 (Selleckchem, Houston, TX, USA) and or decitabine. Cell concentration and viability were assessed using trypan blue dye exclusion test. The number of metabolic-active cells was also determined using intracellular ATP quantitation. For drug combination assay, HMCLs were cultured for 4 days in 96-well flat-bottom microtiter plates in RPMI 1640 medium, 10% FCS, and 2 ng/ml IL-6 (control medium) in the presence of lenalidomide. Cell growth was evaluated by quantifying intracellular ATP amount with a Cell Titer Glo Luminescent Assay (Promega, Madison, WI, USA) using a Centro LB 960 luminometer (Berthold Technologies, Bad Wildbad, Germany).

### Global H3K27me3 and IKZF1 immunofluorescence

After deposition on slides using a Cytospin centrifuge, cells were fixed with 4% PFA, permeabilized with 0.5% Triton in PBS and saturated with 5% bovine milk in PBS. The rabbit anti-H3K27me3 (Active Motif, Rixensart, Belgium, #39156) and anti-IKZF1 (Santa Cruz Biotechnology, Heidelberg, Germany, H-100 sc-3039) antibodies were diluted 1/500 and 1/250 respectively in 5% bovine milk in PBS and deposited on cytospins for 60 min at room temperature. Slides were washed twice, and anti-rabbit alexa 555-conjugated antibodies (diluted 1/500 in 5% bovine milk in PBS) were added for 60 min at room temperature. Slides were washed and mounted with Vectashield and 1% DAPI. Images and fluorescence were captured with a ZEISS Axio Imager Z2 microscope (× 63 objective) (Oberkochen, Germany) and analyzed with Omero (omero.mri.cnrs.fr) server and ImageJ software.

### RNA sequencing

HMCLs were cultured for 4 days without or with 1 μM of EPZ6438. RNA samples were collected as previously described. The RNA sequencing (RNA-seq) library preparation was done with 150 ng of input RNA using the Illumina TruSeq Stranded mRNA Library Prep Kit. Paired-end RNA-seq were performed with Illumina NextSeq sequencing instrument (Helixio, Clermont-Ferrand, France). RNA-seq read pairs were mapped to the reference human GRCh37 genome using the STAR aligner [21]. All statistical analyses were performed with the statistics software R (version 3.2.3; available from https://www.r-project.org) and R packages developed by BioConductor project (available from https://www.bioconductor.org/) [22]. The expression level of each gene was summarized and normalized using DESeq2 R/Bioconductor package [23]. Differential expression analysis was performed using DESeq2 pipeline. *P* values were adjusted to control the global FDR across all comparisons with the default option of the DESeq2 package. Genes were considered differentially expressed if they had an adjusted *P* value of 0.05 and a fold change of 1.5. Pathway enrichment analyses were performed using online curated gene set collection on the Gene Set Enrichment Analysis software (http://software.broadinstitute.org/gsea/msigdb/index.jsp) [24, 25].

### Gene expression profiling and statistical analyses

Gene expression data were normalized with the MAS5 algorithm and analyses processed with GenomicScape (http://www.genomicscape.com) [26] the R.2.10.1 and Bioconductor version 2.5 programs [22]. Gene set expression analysis (GSEA) was used to identify genes and pathways differentially expressed between populations. Univariate and multivariate analysis of genes prognostic for patients’ survival was performed using the Cox proportional hazard model. Difference in overall survival between groups of patients was assayed with a log-rank test and survival curves plotted using the Kaplan-Meier method (Maxstat R package) [27]. The EZ score was built using our previously published methodologies [28]. EZ score is the sum of the Cox beta-coefficients of each of the 15 EPZ6438-deregulated genes with a prognostic value, weighted by ± 1 if the patient MMC signal for a given gene is above or below the Maxstat reference value of this gene (Table 1) [28].

**Table 1:** EZ score genes

Probeset	Name	Maxstat_Cutpoint	Chisq	*P* value	Hazard_Ratio	Prognostic
210841_s_at	NRP2	702	4.3	0.038	1.6	Bad
204364_s_at	REEP1	226	13	0.00028	2	Bad
205551_at	SV2B	53	4.6	0.032	1.5	Bad
43511_s_at	ARRB1	293	3.9	0.047	0.61	Good
211802_x_at	CACNA1G	302	4.4	0.035	0.5	Good
1555480_a_at	FBLIM1	73	5.1	0.024	0.66	Good
207822_at	FGFR1	240	5	0.026	0.51	Good
1552477_a_at	IRF6	111	4.4	0.035	0.55	Good
227297_at	ITGA9	34	13	0.00029	0.51	Good
235560_at	NOVA2	85	3.9	0.049	0.67	Good
228140_s_at	PPP2R2C	131	5.8	0.016	0.55	Good
206628_at	SLC5A1	175	4.3	0.038	0.63	Good
212560_at	SORL1	29	9.1	0.0026	0.52	Good
1559956_at	SYT7	65	5.8	0.016	0.64	Good
213869_x_at	THY1	110	4.2	0.039	0.67	Good

### Cell cycle analysis

HMCLs were cultured in 24-well, flat-bottomed microtiter plates at 10^5^ cells per well in RPMI1640–10% FCS or X-VIVO 20 culture medium with or without IL-6 (3 ng/mL) and EPZ-6438 (Selleckchem). The cell cycle was assessed using DAPI staining (Sigma-Aldrich, Saint-Louis, MO, USA) and cells in the S phase using incubation with bromodeoxyuridine (BrdU) for 1 h and labeling with an anti-BrdU antibody (APC BrdU flow kit, BD Biosciences, San Jose, CA, USA) according to the manufacturer’s instructions.

### Flow cytometry analysis

Cells were fixed for 10 min with Cytofix/Cytoperm (BD Biosciences, San Jose, CA, USA) at 4 °C. The overall expression of MYC, IKZF1, IRF4, and H3K27me3 was evaluated by incubating 10^5^ cells with 5 μLof an alexa 647-conjugated mouse anti-H3K27me3 antibody (Cell Signaling, Danvers, MA, USA, 12158S), alexa 647-conjugated mouse anti-EZH2 antibody (BD Biosciences, 563491) PE-conjugated mouse anti-IKZF1 (BD Biosciences, 564476), rat anti-IRF4 (Biolegend, 646403), rabbit anti-MYC (Cell Signaling, #12189), or anti-Ki67 antibodies in phosphate-buffered saline (PBS) containing 2% FBS at 4 °C for 20 min.

For primary samples, cells were double stained with APC or PE-conjugated anti-CD138 (Beckman-Coulter, Brea, CA, USA). Flow cytometry analysis was done on a Fortessa flow cytometer (BD, Mountain View, CA, USA).

### Study of apoptosis

HMCLs were cultured in 24-well, flat-bottomed microtiter plates at 10^5^ cells per well in RPMI1640–10% FCS or X-VIVO 20 culture medium with or without IL-6 (3 ng/mL), EPZ-6438 (Selleckchem), and QVD. After 8 days of culture, cells were washed twice in PBS and apoptosis was assayed with PE-conjugated Annexin V labeling (BD Biosciences) using a Fortessa flow cytometer (BD).

### H3K27me3-associated genes in MM patients

The list of H3K27me3 associated genes in MM patients was recovered from GEO GSE53215 and used for the development of the EZ score.

### RT-qPCR

RNA was converted to cDNA using the Qiagen QuantiTect Reverse Transcription Kit (Qiagen, Hilden, Germany). The assays-on-demand primers and probes and the TaqMan Universal Master Mix were used according to the user’s manual (Biosystems, Courtaboeuf, France). The measurement of gene expression was performed using the Roche LC480 Sequence Detection System. For each primer, serial dilutions of a standard cDNA were amplified to create a standard curve, and values of unknown samples were estimated relative to this standard curve in order to assess PCR efficiency. Ct values were obtained for *GAPDH* and the respective genes of interest during log phase of the cycle. Gene expression was normalized to that of *b2M* (dCt = Ct gene of interest–Ct *b2M*) and compared with the values obtained for a known positive control using the following formula: 100/2ddCt where ddCt = dCt unknown–dCt positive control.

### Western blot

Cells were lysed in RIPA buffer (Cell Signaling Technology, Beverly, MA, USA) supplemented with 1 mM phenylmethylsulfonyl fluoride immediately before use. Lysates were separated by sodium dodecyl sulfate-polyacrylamide gel electrophoresis (10% gels) and transferred to nitrocellulose membranes using an iBlot® Gel Transfer Device (InVitrogen). Non-specific membrane sites were blocked by incubation at room temperature in 140 mM NaCl, 3 mM KCl, 25 mM Tris-HCl (pH 7.4), 0.1% Tween 20 (tris-buffered saline Tween-20), 5% non-fat milk for 2 h, and then immunoblotted with rabbit polyclonal antibodies against Ikaros (Santa Cruz Biotechnology, Dallas, TX, USA), cMyc (Cell Signaling Technology), or IRF4 (Santa Cruz Biotechnology, Dallas, TX, USA). As a control for protein loading, a mouse monoclonal anti-β-actin antibody (Sigma-Aldrich) was used. The primary antibodies were visualized with peroxidase-conjugated goat anti-rabbit (Sigma-Aldrich) or goat anti-mouse (Jackson ImmunoResearch, West Grove, PA, USA) antibodies and an enhanced chemiluminescence detection system. Western blots were quantified by densitometry using the NIH ImageJ software (National Institutes of Health, Bethesda, MD, USA), and protein levels were normalized according to those of β-actin.

### Chromatin immunoprecipitation followed by sequencing

Cells were cross-linked in formaldehyde at a final concentration of 1% for 8 min. All experiments reagents were included in the Auto iDeal ChIP-seq kit for Histones (Diagenode, Liege, Belgium). Sonication was performed using a Bioruptor Plus sonication devise (Diagenode, Liege, Belgium) under the optimal conditions to shear cross-linked DNA to fragments if 100–300 base pairs in length. ChIP was conducted with the IPStar Compact Automated System (Diagenode, Liege, Belgium). ChIP were performed starting from 1 million cells per IP, using the indirect method in 200 μL final volume. Crossed-linked DNA was incubated 13 h with the antibody (H3K27me3 catalog number C1540195 or H3K4me3 catalog number C15410003) and 3 h with the beads. After 5 min washes, eluates were recovered and reverse cross-linked for 4 h at 65 °C. Samples were treated for 1 h with RNAse at 37 °C, prior to DNA purification with the Auto IPure kit v2 (Diagenode, C03010010). Libraries were performed using NEBNext Ultra Library Prep Kit for Illumina (New England Biolabs). Sequencing was performed with Illumina NextSeq500 technology (Helixio, Clermont-Ferrand, France) using the following parameters: single-read, 50 bp, 40 million reads.

### DNA methylation analysis

Methylation analysis was performed using the Illumina Infinium HumanMethylation450 BeadChip array (HM450K, Illumina Inc.). The microarray raw intensities were preprocessed using the R/Bioconductor package minfi [29].The methylated CpGs overlapping with genes promoter region were extracted using the R package GenomicFeatures [30] implemented in Bioconductor. A CpG is defined as highly methylated when the methylation beta level is above 0.8.

## Results

### PRC2 complex is overexpressed in malignant plasma cells in association with cell cycle deregulation

**Fig. 1:**
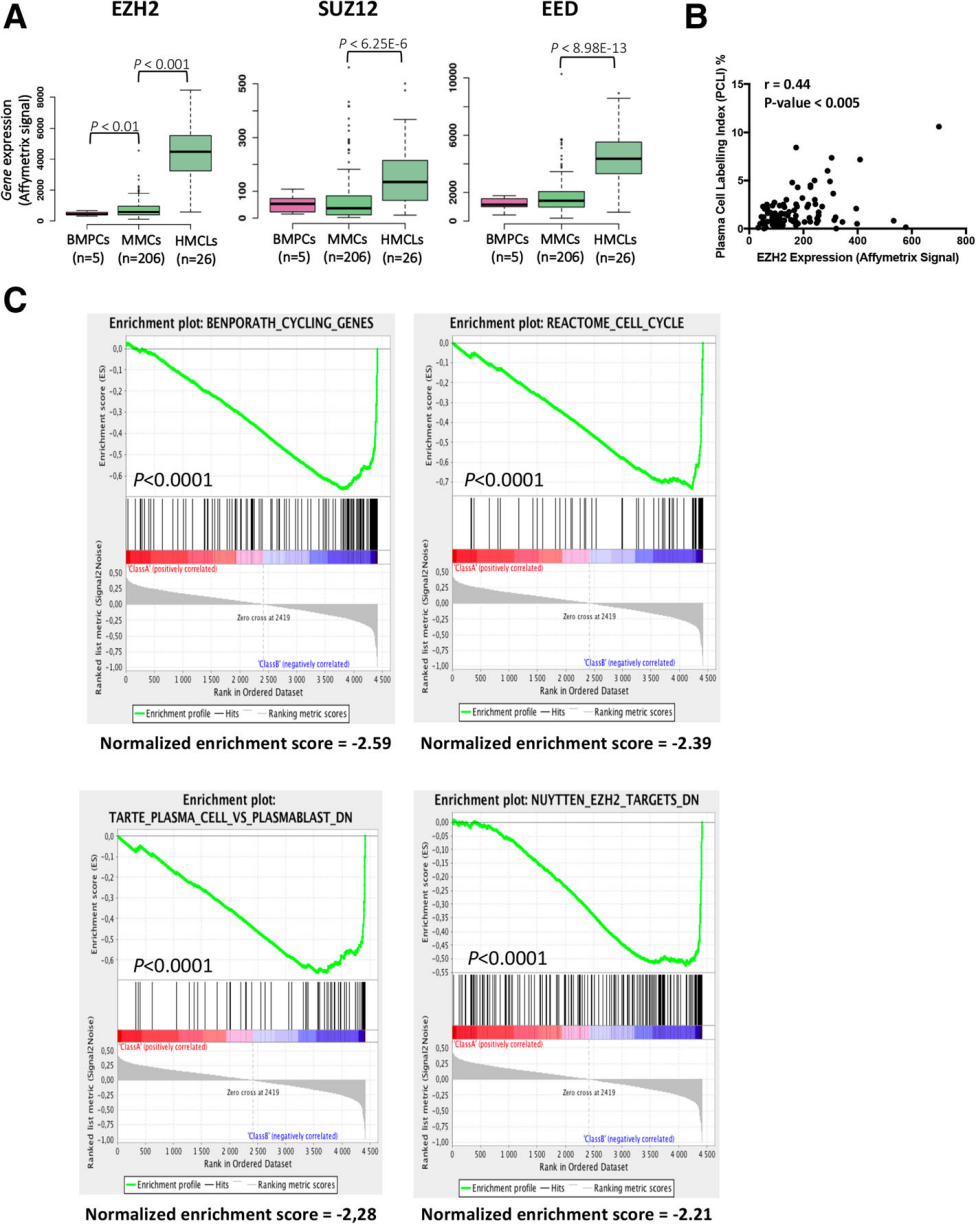
PRC2 complex is deregulated in MM in association with cell proliferation. **a** EZH2, SUZ12, and EED gene expression in BMPCs, patients’ MMCs, and HMCLs. Data are MAS5-normalized Affymetrix signals (U133 plus 2.0 microarrays). Statistical difference was assayed using a *t* test. **b** Correlation between EZH2 expression and malignant plasma cell labeling index. Plasma cell labeling index represents the percentage of malignant plasma cells in S phase of the cell cycle. It was investigated using Brdu incorporation and flow cytometry in 101 patients at diagnosis. **c** Top gene sets significantly associated with high EZH2 expression in MM using GSEA

Using Affymetrix microarrays, we analyzed the expression of PRC2 core genes *EZH2*, *SUZ12*, and *EED* in normal bone marrow plasma cells (BMPCs, *n* = 5), primary myeloma cells from patients (MMCs, *n* = 206), and human myeloma cell lines (HMCLs, *n* = 26). PRC2 core genes are significantly overexpressed in MM cells (Fig. 1a) and *EZH2* expression is significantly correlated with *SUZ12* and *EED* expression (Additional file 1: Figure S1). At the opposite, PRC1 core genes were significantly downregulated in MM cells compared to normal plasma cells underlining polycomb complex deregulation in MM (Additional file 1: Figure S2).

Investigating the prognostic value of *EZH2*, *SUZ12*, and *EED* expression in independent cohorts of previously untreated MM patients, using Maxstat R algorithm [31], only *EZH2* expression was found to be associated with MM patient’s outcome as recently reported [13] (Additional file 1: Figure S3). *EZH2* is significantly overexpressed in patients with del17p and 1q21 gain (Additional file 1: Figure S4A and B). *EZH2* expression is significantly correlated with MMC plasma cell labeling index (PCLI) in a cohort of 101 newly diagnosed patients (*P* < 0.005; Fig. 1b) underlining a link between PRC2 expression and deregulation of cell cycle in MM cells. Furthermore, GSEA analysis of patients with high *EZH2* expression identified a significant enrichment for genes involved in cell cycle, upregulated in proliferating plasmablasts compared to mature BMPCs, and EZH2 targets (*P* < 0.0001) (Fig. 1c).

Altogether, these data underline that PRC2 complex is overexpressed in MMCs in association with a proliferative plasmablastic gene signature and a poor prognosis.

**Fig. 2:**
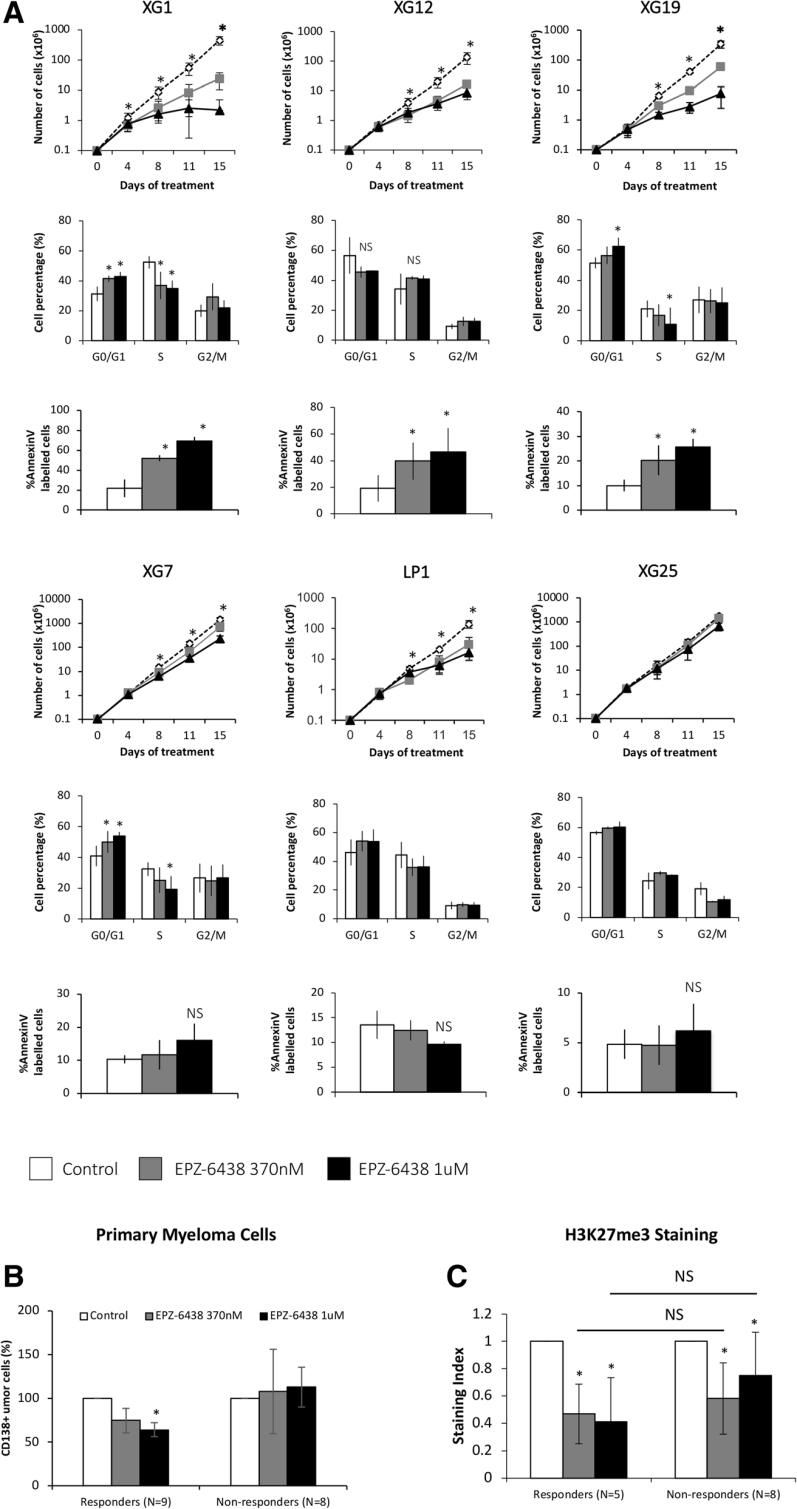
EZH2 inhibition differentially affects MMCs survival. **a** EPZ-6438 treatment leads to MM cell growth inhibition and apoptosis induction. HMCLs were exposed to different doses of EPZ-6438 and cell viability was analyzed after 4, 8, 11, and 15 days. Cells were split, replated, and treated at each time points. Results are the mean absolute cumulated counts ± SD of viable myeloma cells of five independent experiments. Cell cycle of EPZ-6438-treated MM cell lines was analyzed by flow cytometry. Results are representative of four independent experiments. Apoptosis induction was analyzed with Annexin V PE staining by flow cytometry. The shown data are the mean values ± SD of four separate experiments. * indicates a significant difference compared to control cells using a Wilcoxon test for pairs (*P* ≤ 0.05). **b** EZH2 inhibition induces mortality of primary MMCs from patients. At day 8 of culture, the viability and total cell counts were assessed, and the percentage of CD138 viable PC was determined by flow cytometry. Results are median values of the numbers of myeloma cells in the culture wells. **c** Global H3K27me3 status was also assessed by flow cytometry. Results are median values of the H3K27me3 staining index in CD138 viable PC. * indicates a significant difference compared to control cells using a Wilcoxon test for pairs (*P* ≤ 0.05)

To investigate the therapeutic interest of *PRC2* deregulation to target MM cells, XG1, XG12, XG19, LP1, XG25, and XG7 HMCLs were treated with clinically relevant doses of EZH2 inhibitor EPZ-6438 (370 nM and 1 μM) [14, 32]. EPZ-6438 treatment induced a significant decrease of global H3K27me3 in all the HMCLs tested (*P* < 0.01) (Additional file 1: Figures S5 and S6) and inhibited MM cell growth together with proliferation inhibition and apoptosis induction in 3 out of the 6 HMCLs tested (Fig. 2a and Additional file 1: Figure S7). The inhibitory effect appeared at day 6, suggesting that it is mediated by epigenetic reprogramming (Fig. 2a). EZH2 inhibitor induced apoptosis was partially rescued by the QVD pan-caspase inhibitor, suggesting a caspase-dependent mechanism (Additional file 1: Figure S7). LP1 and XG7 were more resistant to EZH2 inhibitor whereas XG25 HMCL was completely resistant. Primary MM cells co-cultured with their bone marrow microenvironment and recombinant IL-6 were also treated with EPZ-6438 as previously described [6]. EZH2 targeting significantly reduced the median number of viable myeloma cells by 35% (*P* = 0.004) in 9/17 patients whereas MM cells of 8 patients were not significantly affected by the EZH2 inhibitor (Fig. 2b and Additional file 1: Figure S8). As described in HMCLs, EPZ-6438 induced a significant global H3K27me3 decrease in all the patients (Fig. 2c). The effect of EZH2 inhibitor was not correlated with EZH2 expression, H3K27me3 and levels in light of the 6 HMCLs and 17 primary MM samples tested. Moreover, UTX/JMJD3 demethylases mutation status did not seem to affect EPZ-6438 efficiency in the tested samples (Additional file 1: Figure S9 and Additional file 2: Table S1).

We therefore conclude that MM cell growth could be affected by PRC2 targeted therapy which presents a therapeutic interest in a subgroup of MM patients.

### Genes deregulated by EZH2 targeted inhibition in myeloma cells

**Fig. 3:**
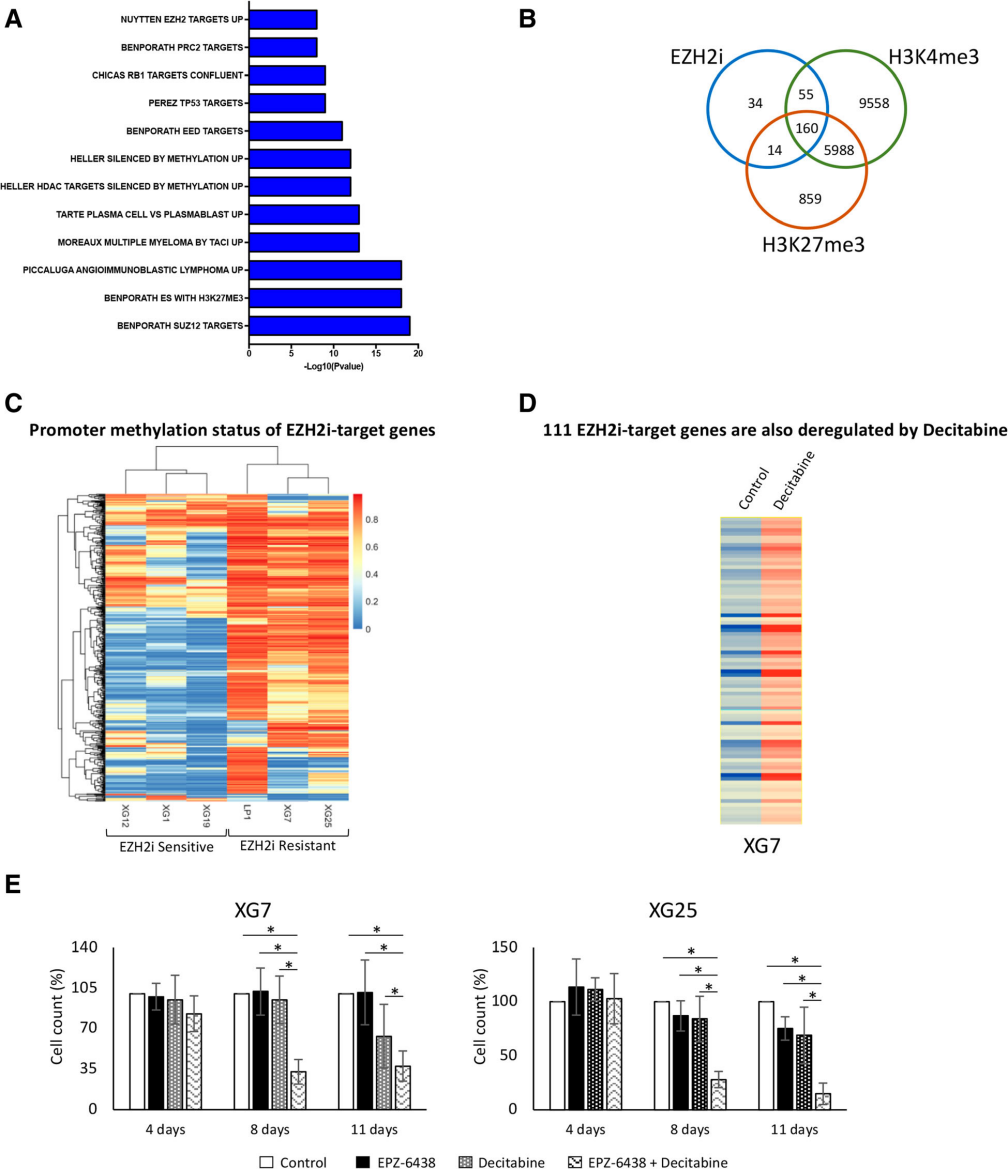
EZH2i-target genes promoters are methylated in EPZ-6438-resistant cells. **a** Molecular signature of EZH2i-target genes in six HMCLs (XG1, XG12, XG19, XG7, LP1, and XG25) was investigated using GSEA Database (all curated gene sets), and relevant pathways were presented (FDR *q* value ≤ 0.05). **b** Venn diagram presenting EZH2i-target genes compared to genes associated with H3K27me3 or H3K4me3 in XG7 HMCL. **c** Methylation status of CpGs overlapping with PRC2 target genes promoter region in EZH2i sensitive and resistant myeloma cell lines. **d** Heatmap presenting 111 EZH2i-target genes that are deregulated by decitabine using Affymetrix U133 plus 2.0 microarrays. **e** HMCLs were exposed to 1 μM of EPZ-6438 and/or 100 nM of decitabine. Cell viability was analyzed by trypan blue assay after 4, 8, and 11 days of treatment. Results are the percentage ± SD of viable myeloma cells of three independent experiments. * indicates a significant difference compared to control using a Wilcoxon test for pairs (*P* ≤ 0.05)

To better understand the molecular mechanisms associated with PRC2 deregulation in MM, six HMCLs were treated with 1 μM of EPZ-6438 for 4 days, and GEP were analyzed using RNA sequencing. Two hundred sixty-three genes are significantly upregulated in EPZ-6438-treated MMC compared to untreated cells (fold change ≥ 2, 1000 permutations, FDR < 0.05, Additional file 3: Table S2). Notably, no gene was significantly downregulated after EPZ-6438 treatment. EPZ-6438-regulated genes are significantly enriched in polycomb target genes, genes enriched in H3K27me3 histone mark, genes described to be associated with DNA methylation in MM, TP53, and RB1 target genes (Fig. 3a and Additional file 4: Table S3). Interestingly, a set of these genes is overexpressed in non-cycling mature normal plasma cells compared to proliferating plasmablasts (Fig. 3a and Additional file 4: Table S3). Among these 263 genes deregulated after EPZ-6438 treatment, 174 were also associated with H3K27me3 mark in XG7 HMCL including 160 bivalent genes characterized by H3K4me3 and H3K27me3 histone marks (Fig. 3b and Additional file 5: Table S4).

### Resistance to EZH2 inhibitor is mediated by DNA methylation of PRC2 target genes

Since PRC2 target genes were associated with a significant enrichment of genes presenting DNA methylation in MM, we analyzed the methylation status at the promoter region of EZH2 inhibitor target genes using 450 k microarrays. Interestingly, 155 out the 263 EZH2 inhibitor target genes were associated with significant CpG methylation in their promoter (Fig. 3c). Furthermore, 111 of these 155 genes were also significantly upregulated after HMCL treatment with decitabine (ratio > 1.5) (Fig. 3d). A significant difference in CpG methylation status of EZH2 inhibitor target genes were identified between sensitive and resistant HMCLs (*P* < 0.01) (Fig. 3c). Interestingly, combination of a sublethal dose of decitabine (100 nM, IC10) with EPZ-6438 (1 μM) allowed to sensitize XG7 and XG25 EPZ-6438-resistant HMCLs. After 8 and 11 days of treatment, we observed a significant cell growth inhibition in both cell lines (67.4% and 62.4% respectively, *P* < 0.01 in XG7, and 72.2% and 85.2% respectively in XG25), compared to decitabine or EPZ-6438 used alone (*P* < 0.05) (Fig. 3e).

These data demonstrate that PRC2 target genes could be associated with DNA methylation underlying an overlap between epigenetic silencing mechanisms on potent key MM tumor suppressor genes.

### PRC2 inhibition sensitizes myeloma cells to lenalidomide

**Fig. 4:**
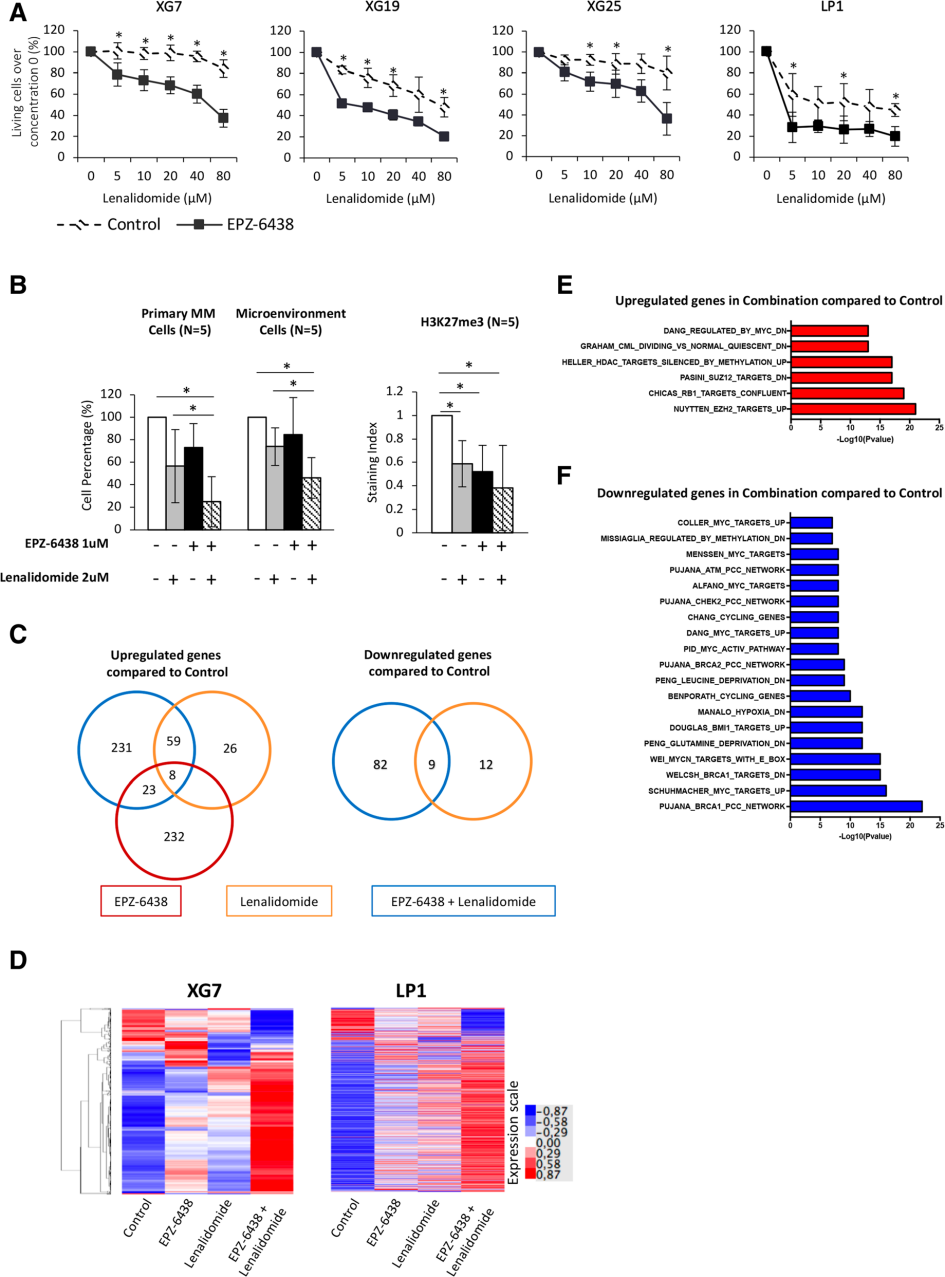
EZH2 inhibition sensitizes HMCLs and primary MMCs to lenalidomide. **a** HMCLs were treated 4 days with 1 μM EPZ-6438 and then cultured 4 days with graded lenalidomide concentrations. Data are mean values ± standard deviation (SD) of five experiments. * indicates a significant difference compared to control cells using *t* test (*P* ≤ 0.05). **b** After a 4-day pre-treatment with EPZ-6438 (1 μM), mononuclear cells from five patients with MM were treated for 4 days with 2 μM lenalidomide. The viability and total cell counts were assessed, and the percentage of CD138 viable PC and non-malignant bone marrow cells was determined by flow cytometry. Results are median values of the numbers of myeloma cells in the culture wells. Results were compared with a Wilcoxon test for pairs. **c** EPZ-6438, lenalidomide, and EPZ-6438/lenalidomide combination deregulated genes in MM. XG7 and LP1 HMCLs were treated with 2 μM lenalidomide (2 days) with (combination) or without (lenalidomide) prior 4 days-treatment with 1 μM EPZ-6438. Venn diagram showing overlap of genes deregulated by EPZ-6438, lenalidomide, or EPZ-6438/lenalidomide combination. **d** Heatmaps of RNA-seq analysis presenting expression profiles of EPZ-6438, lenalidomide, or combination target genes in XG7 and LP1 HMCLs. **e**, **f** Molecular signature of EPZ-6438 and lenalidomide combination deregulated genes compared to control in XG7 and LP1 was investigated using GSEA Database (all curated gene sets), and relevant pathways were presented (FDR *q* value ≤ 0.05)

We investigated whether EZH2 inhibition could enhance the anti-myeloma activity of melphalan, bortezomib, and lenalidomide treatment. EPZ-6438 pretreatment does not significantly enhance the effect of melphalan and bortezomib treatment on MMCs in vitro (data not shown). However, EPZ-6438 pretreatment significantly sensitized HMCLs to lenalidomide treatment. Treatment for 4 days with 1 μM of EPZ-6438 prior to lenalidomide treatment significantly enhanced toxicity on LP1 and XG19 HMCLs (*P* < 0.05) (Fig. 4a). Furthermore, EZH2 inhibition was able to overcome lenalidomide resistance in XG7 and XG25 cell lines (Fig. 4a). These data strongly suggest that combination of EZH2 inhibitor and IMiDs could be of therapeutic interest in MM.

These results were validated using primary MMCs from patients [6, 28]. The median percentage of viable MMCs was reduced of 43.4%, 26.9%, and 75.1% when cells were treated with lenalidomide, EPZ-6438, or the combination EPZ-6438/lenalidomide respectively (*P* = 0.003; *n* = 5) (Fig. 4b). Global H3K27me3 levels showed a tendency toward decrease under combination treatment, although the difference was not significant.

### PRC2 targeting combined with Lenalidomide induced MM cell transcriptional reprogramming

Using RNA sequencing GEP data, we then compared MM cell lines treated by EPZ-6438, lenalidomide, or EPZ-6438/lenalidomide combination. Thirty-one genes were commonly upregulated by the EPZ-6438 and EPZ-6438/lenalidomide combination (Fig. 4c). Sixty-seven percent of the genes deregulated by lenalidomide in MM are also deregulated by the EPZ-6438/lenalidomide combination (Fig. 4c and Additional file 6: Table S6). Furthermore, EPZ-6438/lenalidomide induced a significantly higher deregulation of EPZ-6438 or lenalidomide target genes including *PARP9*, *RGS1*, *DKK1*, *SEZ6L2*, *CAV2*, *ANTXR1*, *EMP1*, and *ANXA1* (ratio > 1.5, *P* < 0.05; Fig. 4d and Additional file 6: Table S6 and Additional file 7: Table S7).

Seventy-six percent of EPZ-6438/lenalidomide deregulated genes were not affected by EPZ-6438 or lenalidomide alone, suggesting that EZH2 inhibitor and IMiDs combination could modulate the expression of a specific set of genes. Among the 231 genes significantly upregulated uniquely after EPZ-6438/lenalidomide treatment, we found a significant enrichment of PRC2 and RB1 target genes, of genes downregulated by MYC, and genes silenced by DNA methylation (Fig. 4e and Additional file 8: Table S8). GSEA analysis of the 82 specifically downregulated genes by EPZ-6438/lenalidomide revealed a significant enrichment of MYC target genes in association with genes involved in proliferation and replicative stress response (Fig. 4f and Additional file 8: Table S8). Altogether, these data indicate that EPZ-6438/lenalidomide induced a significant upregulation of PRC2, RB1, and DNA methylation target genes in association with a significant downregulation of MYC target genes and cell proliferation gene program.

**Fig. 5:**
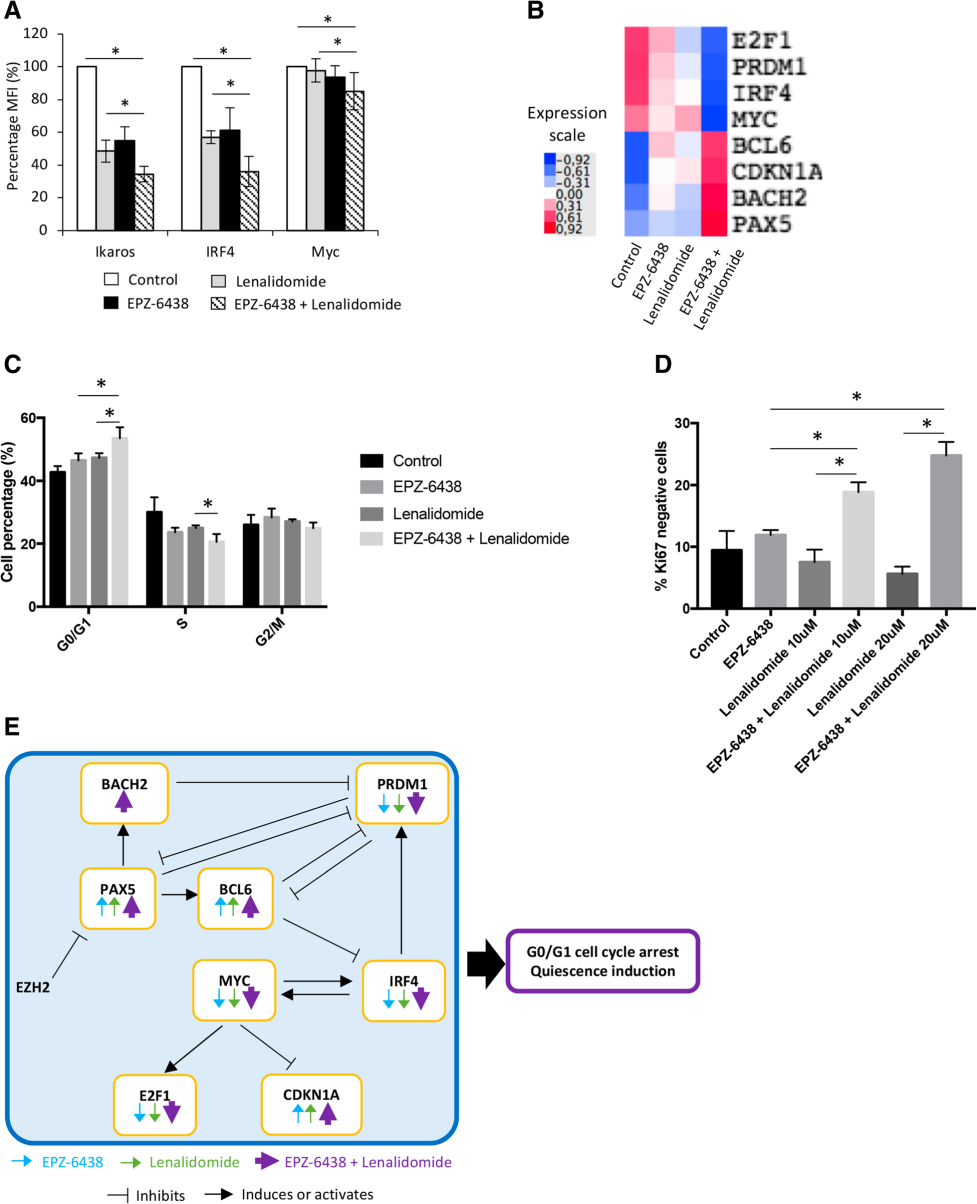
EPZ-6438/lenalidomide combination targets key B cell transcription factors and induces MMCs quiescence. **a** The expression of Ikaros (IKZF1), c-Myc, and IRF4 of HMCLs was evaluated by flow cytometry in XG7 after EPZ-6438 (1 μM) and/or lenalidomide (2 μM) treatment using PE-conjugated anti-IKZF1, anti-MYC and anti-IRF4 mAb, and isotype matched PE-conjugated mAb. Data are mean values ± standard deviation of three experiments. **b** Heatmap presenting RNA-seq GEP expression of E2F1, PRDM1, IRF4, MYC, BCL6, CDKN1A, BACH2, and PAX5 in XG7 HMCL treated with EPZ-6438 (1 μM) and/or lenalidomide (2 μM). **c** XG7 cell cycle was analyzed, after EPZ-6438 (1 μM) and/or lenalidomide (2 μM) treatment, by flow cytometry using DAPI, BrdU incorporation, and labeling with an anti-BrdU antibody. Results are representative of four independent experiments. * indicates a significant difference compared to control cells using a paired *t* test (*P* ≤ 0.05). **d** The percentage of Ki67 negative cells of EPZ-6438 and/or lenalidomide-treated XG7 HMCL was analyzed by flow cytometry using anti-Ki67 antibody. Data are the mean values ± SD of three separate experiments. * indicates a significant difference compared to control cells using a paired *t* test (*P* ≤ 0.05). **e** Model of EPZ-6438/lenalidomide combination action in MMCs

IMiDs promote Ikaros and Aiolos transcription factors binding to the E3 ubiquitin ligase cereblon (CRBN) leading to their ubiquitination and proteasomal degradation [33]. Ikaros and Aiolos degradation is associated with a downregulation of interferon regulatory factor 4 (IRF4) and MYC which, in turn, reduces MMCs survival [34]. Investigating the effect of EPZ-6438/lenalidomide combination in XG7 and LP1 MMCs, we found that Ikaros (IKZF1), IRF4, and MYC protein levels were significantly decreased by the combination treatment (65.5%, 63.9%, and 14.8% respectively) compared with lenalidomide (51.5%, 43% and 2.2%) or EPZ-6438 (45.2%, 38.7%, and 6.2%) alone (Fig. 5a). These data were validated by western blot and immunofluorescence (Additional file 1: Figures S10 and S11). Furthermore, EPZ-6438/lenalidomide combination strongly upregulated *PAX5*, *BACH2*, and *BCL6* B cell transcription factors in association with downregulation of PRDM1 and IRF4 key plasma cell transcription factor (Fig. 5b). These data were validated by q-RT-PCR (Additional file 1: Figure S12). Interestingly, ChIP-seq analysis showed the bivalence of *PAX5* gene. *PAX5* expression could be directly regulated by EZH2 through H3K27me3 (Additional file 1: Figure S13). EPZ-6438/lenalidomide mediated transcriptional deregulation resulted in significant XG7 HMCL proliferation inhibition and quiescence induction characterized by a significant increase in the percentage of G0/G1 cells and ki67 negative cells together with E2F1 downregulation and CDKN1A induction (Fig. 5c–e).

### EZ-GEP-based score allows to predict sensitivity of MMCs to EZH2 inhibitor treatment

**Fig. 6:**
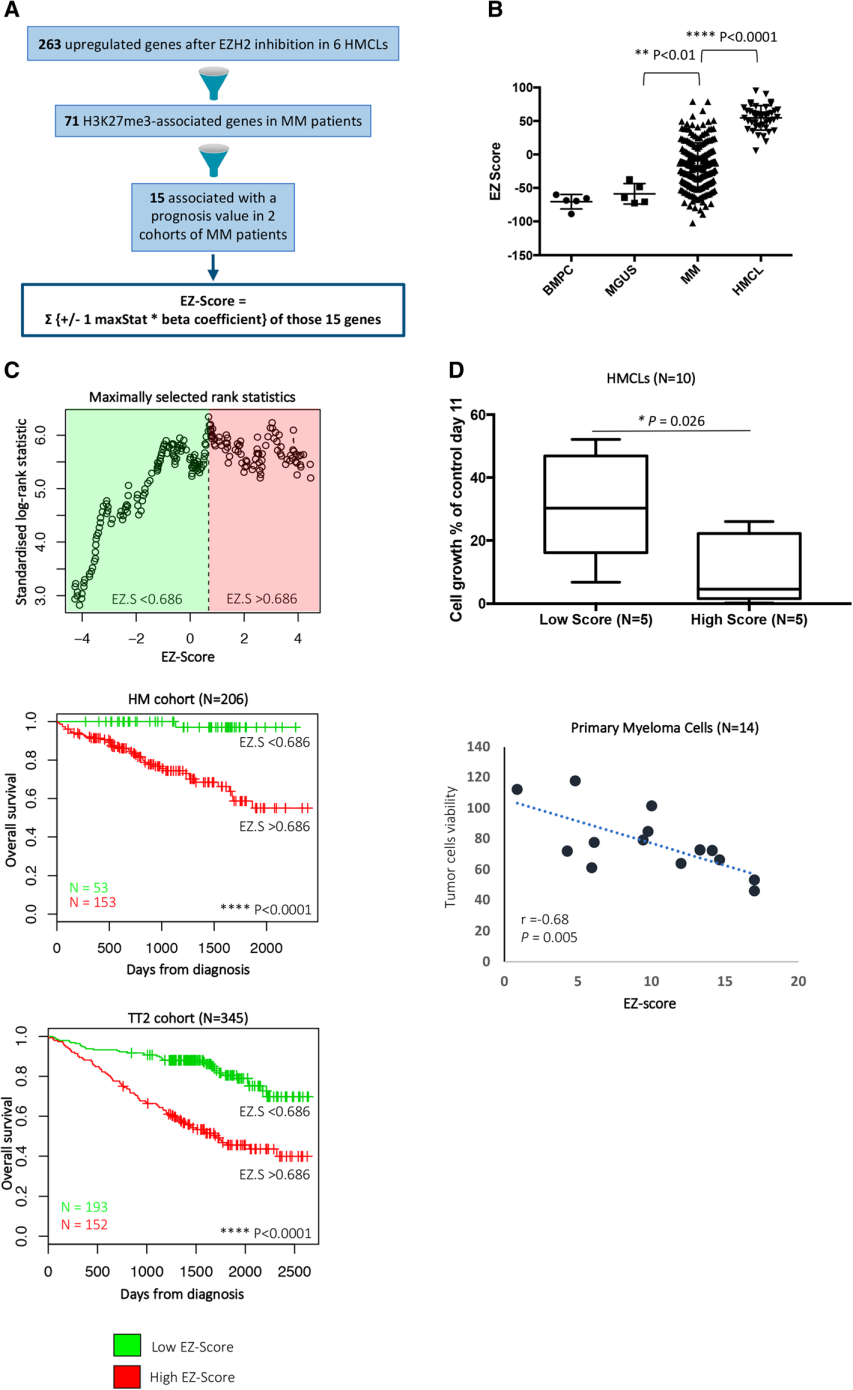
EZ score can predict for sensitivity of poor prognosis MM patients to EZH2i. **a** Chart explaining the process of the EZ score creation. **b** EZ score in normal BMPCs (*N* = 7), in premalignant PCs of patients with MGUS (*N* = 5), in MMCs of patients (*N* = 206) and in HMCLs (*N* = 40). Results were compared with a Student’s *t* test. **c** Prognostic value of the EZ score in MM. Patients of the HM cohort were ranked according to increased EZ score and a maximum difference in OS was obtained with EZ score of 0.686 splitting patients into high-risk (*N* = 153) and low-risk (*N* = 53) groups. EZ score also had a prognostic value of an independent cohort of 345 patients (UAMS-TT2 cohort). The parameters to compute the EZ score of patients of UAMS-TT2 cohort and the proportions delineating the two prognostic groups were those defined with HM cohort. **d** HMCLs with high EZ score (*N* = 5) exhibit significant higher EPZ-6438 sensitivity compared with HMCLs with low EZ score (*N* = 5). Data are mean values ± standard deviation of five experiments determined. **e** EZ score predicts for EPZ-6438 sensitivity of primary myeloma cells of patients. Mononuclear cells from tumor samples of 14 patients with MM were cultured for 8 days in the presence of IL-6 (2 ng/ml) with or without 1 μM EPZ-6438. At day 8 of culture, the count of viable MMCs was determined using CD138 staining by flow cytometry

Since the sensitivity of MMC to EZH2 inhibition is heterogeneous, we searched to define a biomarker allowing the identification of MM patients that could benefit from EZH2 inhibitor treatment. We built a GEP-based score (EZ score), using the prognostic information of 15 genes deregulated by EPZ-6438 and associated with H3K27me3 and a prognostic value in newly diagnosed MM patients (Fig. 6a and Additional file 9: TableS5). The EZ score is defined by the sum of the beta coefficients of the Cox model for each prognostic gene, weighted by − 1 according to the patient MMC signal above or below the probe set Maxstat value as previously described [16]. EZ score levels in normal, premalignant, or malignant plasma cells are displayed in Fig. 6b. MMCs of patients had a significantly higher EZ score than normal BMPCs or plasma cells from MGUS patients (*P* < 0.01). HMCLs present an even higher EZ score compared with primary MMCs (*P* < 0.001; Fig. 6b), demonstrating an association between EZ score and the progression of the disease.

Using patient’s HM and UAMS-TT2 cohorts, the EZ score had prognostic value when used as a continuous variable or by splitting patients into two groups using the Maxstat R function [16]. The EZ score split patients in a high-risk group (EZ score > 0.686) and a low-risk group (EZ score < 0.686) in the HM and UAMS-TT2 cohorts (*P* < 0.0001) (Fig. 6c).

We then sought to identify whether the EZ score could predict for the sensitivity of HMCLs to EPZ-6438. Using our large cohort of HMCLs [15], we analyzed the response of five HMCLs with the highest EZ score and five HMCLs with the lowest EZ score to EZH2 inhibitor treatment. The five HMCLs with the highest EZ score exhibit a significant 20-fold median higher sensitivity to EPZ-6438 compared with the five HMCLs with low EZ score (*P* = 0.04) (Fig. 6d).

To determine whether the EZ score could predict the sensitivity of primary MMCs to EPZ-6438, we analyzed the correlation between its toxicity on MMCs and the EZ score value in a panel of 14 patients. Primary MMCs were cultured together with their bone marrow environment, recombinant IL-6, and 1 μM of EPZ-6438 for 8 days. As identified in HMCLs, a significant correlation between EZ score and EZH2 inhibitor activity on primary MMCs was observed (*r* = − 0.68; *P* = 0.005) (Fig. 6e). A high EZ score value is associated with a higher toxicity of EZH2 inhibitor in MMCs. The EZ score allows the identification of a subgroup of MM patients with a poor outcome that could benefit from EZH2 inhibitor treatment.

## Discussion

Our results underline a significant deregulation of PRC1 and PRC2 complex in MM and extend recent studies which pointed out a role of EZH2 in MM biology [7–11, 13]. We demonstrated that PRC2 deregulation is associated with cell cycle deregulation and a proliferative plasmablastic gene expression signature. Our data suggest that PRC2 deregulation could support MM physiopathology, dissemination, and progression. EZH2 inhibitors have been reported to induce expression of miRNA and decrease expression of oncogenes [12, 35]. PRC2 targeting, using EZH2 inhibitor, represents a potent therapeutic strategy in a subgroup of MM patients resulting in a significant induction of polycomb target genes, genes associated with DNA methylation, TP53, and RB1 target genes. The analysis of genes deregulated by EED inhibitor or by knock down of other PRC2 component will be of interest to define the PRC2 specific target genes and the EZH2i unspecific targets. As reported in diffuse large B cell lymphoma [36], we found that H3K27me3 global levels are potently reduced after EPZ-6438 treatment in all HMCLs and primary MM samples tested. However, no link with EZH2 inhibitor sensitivity was identified. Even if UTX loss was recently reported to sensitize MM cell lines to EZH2 inhibition [35], neither EZH2 gene expression nor UTX mutation status was predictive of MM cell response to PRC2 targeting in our collection of MM cell lines representative of molecular heterogeneity or in the primary samples tested. However, analyses of a higher number of samples will be important to provide more significant conclusions. However, we identified a significant overlap between H3K27me3 and DNA methylation of EPZ-6438 target genes in MM cells resistant to EZH2 inhibitor. These two epigenetic repression systems are mechanistically linked. Indeed, EZH2, as part of PRC2 complex, is required for its target gene promoter methylation [37]. These data underline that PRC2 target genes could comprise key MM tumor suppressor genes silenced by different epigenetic silencing. Interestingly, sublethal doses of DNMTi allow to sensitize EPZ-6438-resistant MM cell lines to EZH2 inhibitor. A major stake remains the identification of biomarkers that can quickly identify the subset patients who could benefit from EZH2 inhibitor treatment.

We developed an EZ score based on 15 EPZ-6438 target genes associated with H3K27me3 and prognostic value in primary MMCs. Our results demonstrate that the EZ score allows the identification of MM patients with a high EZ score value, an adverse prognosis and who could benefit from treatment with EZH2 inhibitors. The expression of 12 out of the 15 genes building the EZ score is associated with favorable outcome in MM. However, their biological functions in MM remain to be characterized.

Several of those genes are known tumor suppressors that are hypermethylated in solid cancers (*CACNA1G*, *IRF6*, *ITGA9*, and *PPP2R2C*) and used as biomarkers related to adverse outcome [38–51]. In MM, *SORL1*, a member of the low-density lipoprotein receptor family, is significantly hypermethylated at relapse compared to diagnosis in MM [52, 53]. *SLC5A1* encodes a member of the sodium-dependent glucose transporter (SGLT) family. *SLC5A1* is a good prognosis biomarker in cervical tumors. *SLC5A1* activation through MAP17 increases ROS production [54].

*FBLIM1* is a widely expressed protein presenting a role in cellular shape modulation, motility, and differentiation [55]. Interestingly, *FBLIM1* expression is reduced in breast cancer and has also been shown to sensitize glioma cells to cisplatin-induced apoptosis [56, 57]. Members of beta-arrestin such as ARRB1 participate in the sensitization/desensitization of G-protein-coupled receptors. The loss of ARRB1 is related to a poor outcome in NSCLC patients [58]. ARRB1 can regulate apoptosis and DNA repair, and its overexpression induced DNA damage in NSCLC cells [59]. THY1 (CD90) is a cell surface glycoprotein involved in cell adhesion and cell communication. In nasopharyngal carcinoma, THY1 is poorly expressed due to its promoter hypermethylation. THY1 overexpression in this cell type induces a G0/G1 cell cycle arrest [60]. These data emphasize that EZH2 inhibitor induces transcriptional activation of potential MM tumor suppressor genes.

Given that monotherapy remains inefficient in MM, a particular association of drugs is usually used to treat MM disease. Unfortunately, despite rising advances in drug development and patient care, MM patients often relapse [61, 62]. By combining EPZ-6438 with MM conventional drugs, we uncovered its synergistic activity with lenalidomide. Furthermore, pre-treatment of MMCs with EZH2 inhibitor was able to overcome lenalidomide resistance (Fig. 4). Transcriptome analysis of cells treated with EPZ-6438/lenalidomide showed a combination-specific deregulated set of genes enriched in GSEA signature related to MYC targeting and genes involved in proliferation and replicative stress response. Furthermore, PRC2 inhibition and most significantly EPZ-6438/lenalidomide combination upregulated PAX5, BACH2, and BCL6 B cell transcription factors. Recently, EZH2i have been shown to induce BCL6 expression in MM cell lines in association with cell death [35]. PAX5, BCL6, and BACH2 repress PRDM1 plasma cell transcription factor. PRDM1 is known to repress BCL6 and PAX5 [63–66], but epigenetic mechanisms may play a major role in B cell transcription factor silencing during plasma cell differentiation as underlined by the bivalent domain of PAX5, including H3K4me3 and H3K27me3 marks, identified in MM cells (Additional file 1: Figure S13). Accordingly, EPZ-6438/lenalidomide treatment resulted in downregulation of PRDM1 and IRF4 expression. By binding to cereblon (CRBN), the IMiD enhances Ikaros and Aiolos proteosomal degradation, thus leading to IRF4 and MYC downregulation [33]. These two factors have been shown to be critical for MMCs survival and disease progression [34]. Our data revealed that EPZ-6438 and lenalidomide combination interfere with MYC transcriptional activity and significantly decreased Ikaros, IRF4, and MYC expression compared with lenalidomide alone. Finally, we discovered in this study that EZH2 inhibition and IMiDs activity act synergistically to alter gene transcription in MM cells, specifically targeting MM oncogenes and cell cycle genes. The deregulation of B and plasma cell transcription factors mediated by PRC2 targeting and IMiDs results in a significant shift between proliferation and quiescence with a significant increase in the percentage of ki67 negative MM cells and a significant induction of quiescent cell features including repression of E2F1 and higher level of p21 Cdk inhibitor [67–70]. Since MYC activates E2F expression and represses p21 [69], this reprogramming may be explained by the dual targeting of MYC-IRF4 axis by EPZ-6438/lenalidomide combination.

## Conclusion

Altogether, our data demonstrate a deregulation of PRC profiles in MM cells contributing to malignant phenotype. The transcriptional deregulation mediated by PRC2 represent a therapeutic target in MM and a synergy was identified with IMiDs. PRC2 targeting in association with IMiDs could have a therapeutic interest in high-risk MM patients characterized by high EZ score values, reactivating B cell transcription factors and tumor suppressor genes.

## Additional files

Additional file 1: **Figure S1.** EZH2 expression is correlated with EED and SUZ12 expression. Figure S2 PRC1 members are significantly downregulated in primary MM cells compared with normal bone marrow plasma cells. Figure S3 EZH2 is associated with a poor prognosis in MM. Figure S4 Association between EZH2 expression and patients’ genetic abnormalities. Figure S5 EPZ-6438 leads to H3K27me3 loss in HMCLs. Figure S6 EPZ-6438 leads to H3K27me3 loss in HMCLs. Figure S7 EPZ-6438-induced apoptosis is partially rescued by QVD pan caspase inhibitor. Figure S8 EZH2 inhibition induces mortality of primary MM cells from patients. Figure S9 Correlation between EZH2 expression/H3K27m3 staining and drug response to EZH2 inhibitor in HMCLs and patients. Figure S10 IKZF1 protein level decreases after HMCLs were treated with EPZ-6438 and lenalidomide combination. Figure S11 Lenalidomide targets protein levels after treatment. Figure S12 B cell transcription factors mRNA expression after treatment. Figure S13 PAX5 is a bivalent gene in XG7 HMCL. (PDF 21322 kb)

Additional file 2: **Table S1.** HMCLs molecular characteristics (XLSX 15 kb)

Additional file 3: **Table S2.** EPZ-6438 regulated genes in HMCLs (XLSX 32 kb)

Additional file 4: **Table S3.** GSEA signature enrichment of the 264 EPZ-6438 target genes (XLSX 18 kb)

Additional file 5: **Table S4.** EZH2i target genes are mostly bivalent in XG7 HMCLs (XLSX 10 kb)

Additional file 6: **Table S6.** 67 Lenalidomide + combo upregulated genes (XLSX 17 kb)

Additional file 7: **Table S7.** 31 EPZ-6438 + combo upregulated genes (XLSX 11 kb)

Additional file 8: **Table S8.** Lenalidomide+EPZ-6438-regulated genes associated with GSEA signatures (XLSX 75 kb)

Additional file 9: **Table S5.** H3K27me3-associated and EPZ-6438-regulated genes (XLSX 12 kb)

## Abbreviations

DNMTi: DNA methyltransferases inhibitor
EZH2: Enhancer of zeste homolog 2
EZH2i: EZH2 inhibitor
HMCL: Human myeloma cell line
IMiDs: Immunomodulatory drugs
MM: Multiple myeloma
MMC: Multiple myeloma cells
PRC: Polycomb repressive complex
