# Supplementary material for: PRC2 targeting is a therapeutic strategy for EZ score defined high-risk multiple myeloma patients and overcome resistance to IMiDs
Source: Clin Epigenetics. 2018 Oct 3;10:121. doi: 10.1186/s13148-018-0554-4 (PMC6171329; doi:10.1186/s13148-018-0554-4)
Supplement: Supplementary file 1 — Figure S1. EZH2 expression is correlated with EED and SUZ12 expression. Figure S2 PRC1 members are significantly downregulated in primary MM cells compared with normal bone marrow plasma cells. Figure S3 EZH2 is associated with a poor prognosis in MM. Figure S4 Association between EZH2 expression and patients’ genetic abnormalities. Figure S5 EPZ-6438 leads to H3K27me3 loss in HMCLs. Figure S6 EPZ-6438 leads to H3K27me3 loss in HMCLs. Figure S7 EPZ-6438-induced apoptosis is partially rescued by QVD pan caspase inhibitor. Figure S8 EZH2 inhibition induces mortality of primary MM cells from patients. Figure S9 Correlation between EZH2 expression/H3K27m3 staining and drug response to EZH2 inhibitor in HMCLs and patients. Figure S10 IKZF1 protein level decreases after HMCLs were treated with EPZ-6438 and lenalidomide combination. Figure S11 Lenalidomide targets protein levels after treatment. Figure S12 B cell transcription factors mRNA expression after treatment. Figure S13 PAX5 is a bivalent gene in XG7 HMCL. (PDF 21322 kb) [file 13148_2018_554_MOESM1_ESM.pdf]

Supplementary Figures

Supplementary Figure S1

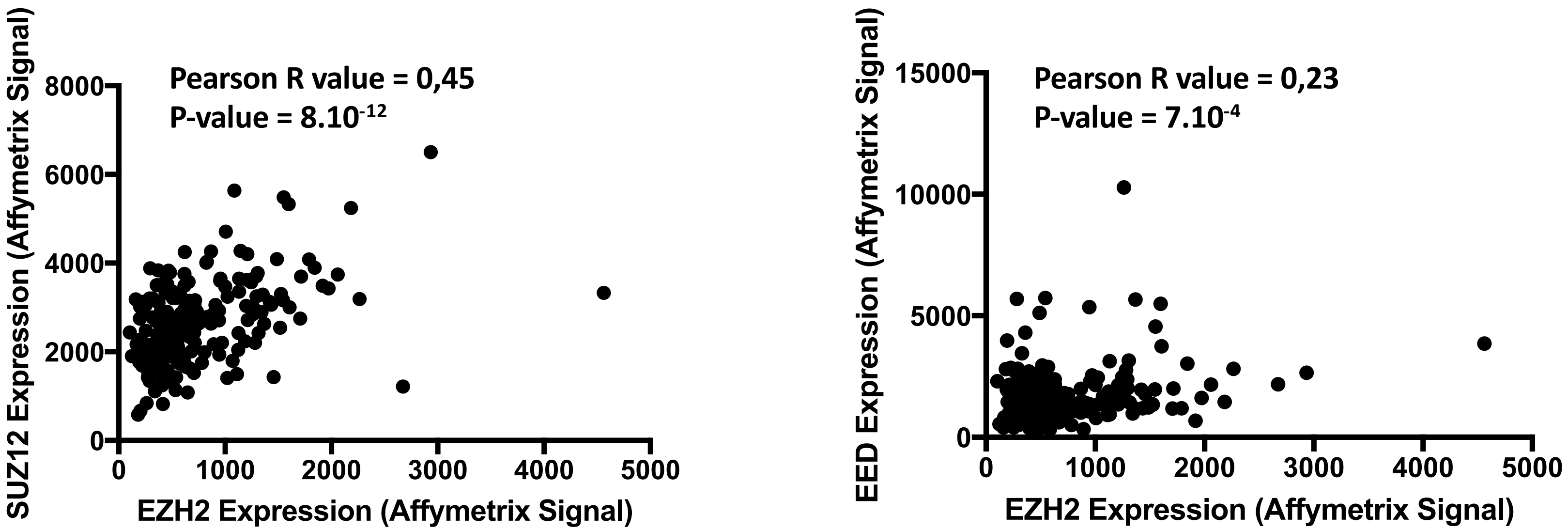

EZH2 expression is correlated with EED and SUZ12 expression

Gene expression was determined using Heidelberg-Montpellier cohort (Affymetrix data). A Pearson correlation test was performed to compare SUZ12 or EED to EZH2 expression in patients MM cells.

Supplementary Figure S2

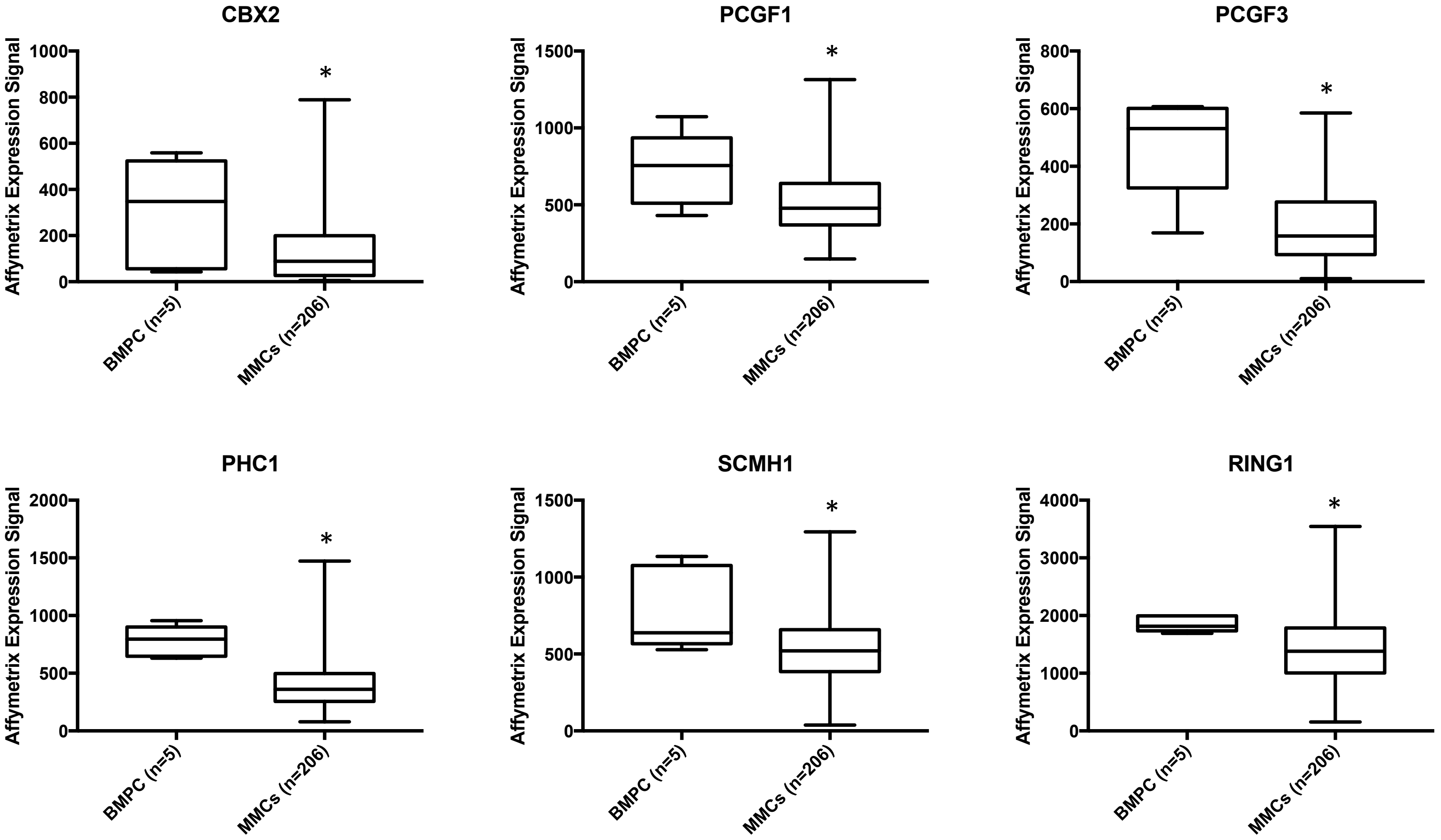

PRC1 members are significantly downregulated in primary MM cells compared with normal bone marrow plasma cells

CBX2, PCGF1, PCGF3, PHC1, SCMH1 and RING1 gene expression in BMPCs, patients' MMCs and HMCLs. Data are MAS5-normalized Affymetrix signals (U133 plus 2.0 microarrays). Statistical difference was assayed using a t-test.

Supplementary Figure S3

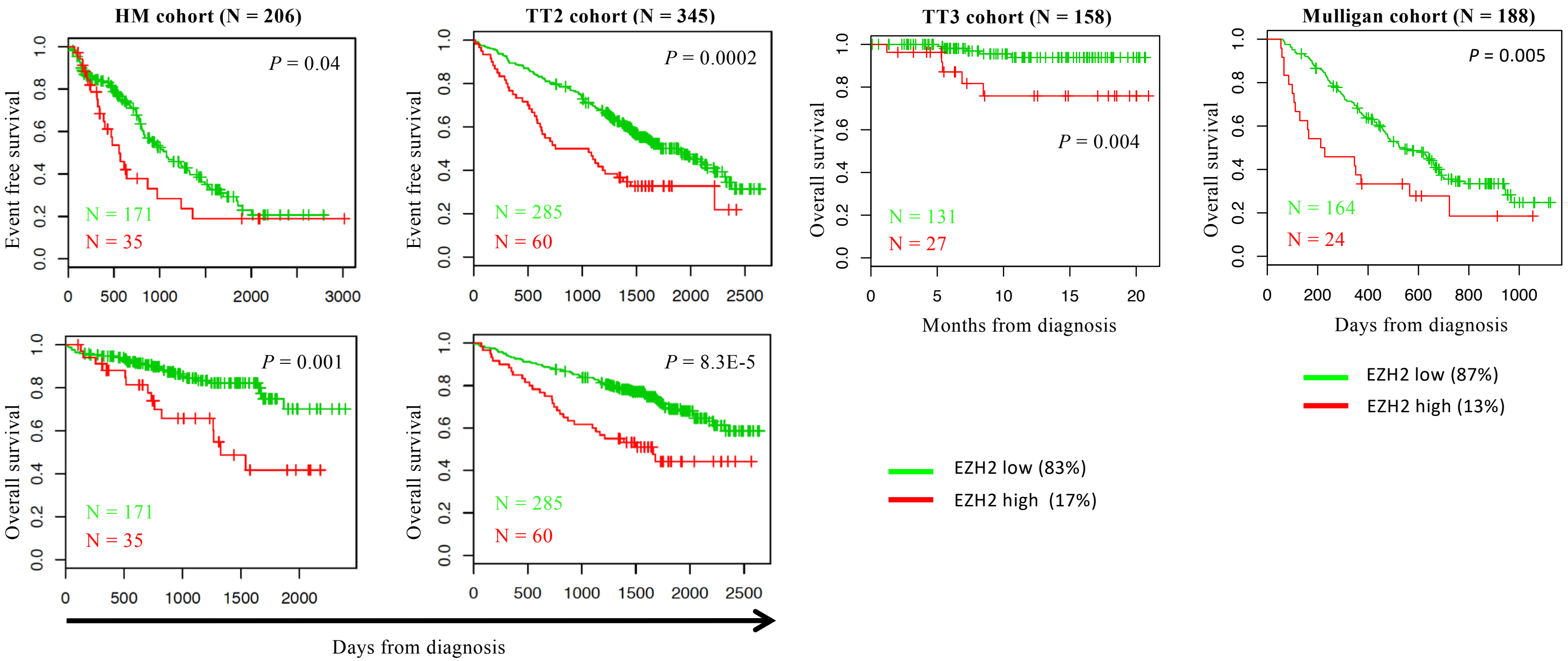

EZH2 is associated with a poor prognosis in MM.
Lower event free and overall survival of newly diagnosed MM patients (HM cohort, N=206) who's MMCs highly expressed EZH2 gene. The splitting of the patients into two groups according to EZH2 expression in MMCs was done using the Maxstat algorithm.
High EZH2 expression is associated with a poor prognosis (EFS and OS) in an independent cohort of 345 patients (UAMS-TT2), in UAMS-TT3 cohort (N=158) and in a cohort a patients at relapse treated by bortezomib monotherapy (Mulligan cohort, N=188)

Supplementary Figure S4

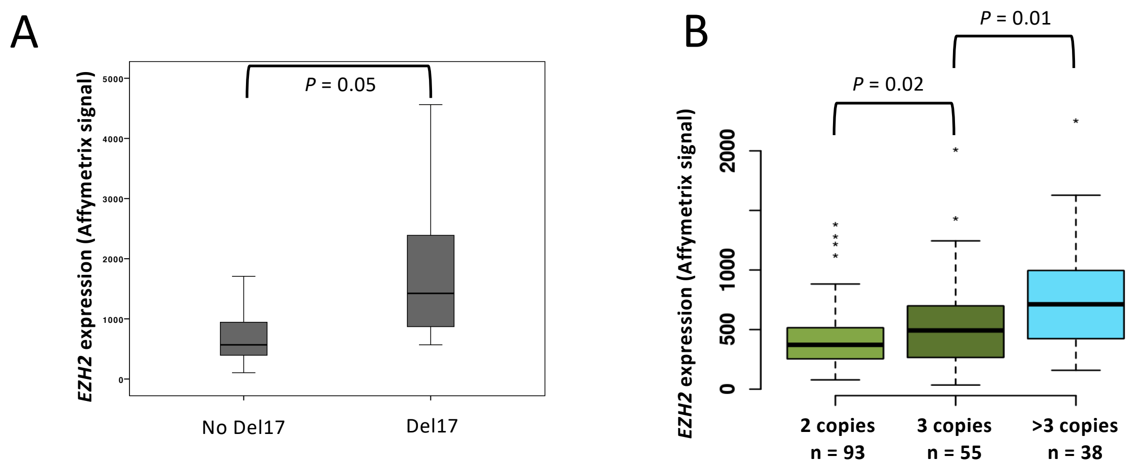

Association between EZH2 expression and patients' genetic abnormalities. Interphase-FISH-analysis was performed on CD138-purified plasma cells of 153 to 169 patients of the HM cohort, depending on the gene abnormality. Statistical significance was tested using a student t test.

Supplementary Figure S5

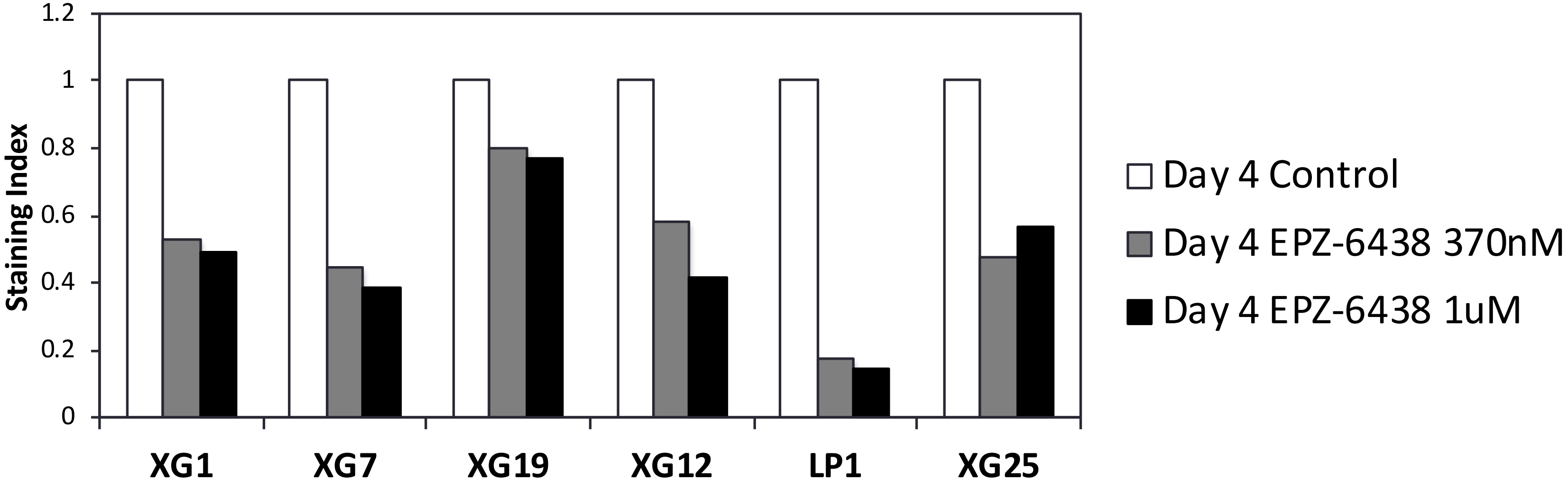

EPZ-6438 leads to H3K27me3 loss in HMCLs. The H3K27me3 status of HMCLs after EPZ-6438 treatment (370nm and 1µM) was analyzed by flow cytometry using AlexaFluor-647 (AF-647)-conjugated anti-H3K27me3 mAb and isotype matched AF-647-conjugated mAb (control). The relative fluorescence was represented by the staining index.

Supplementary Figure S6

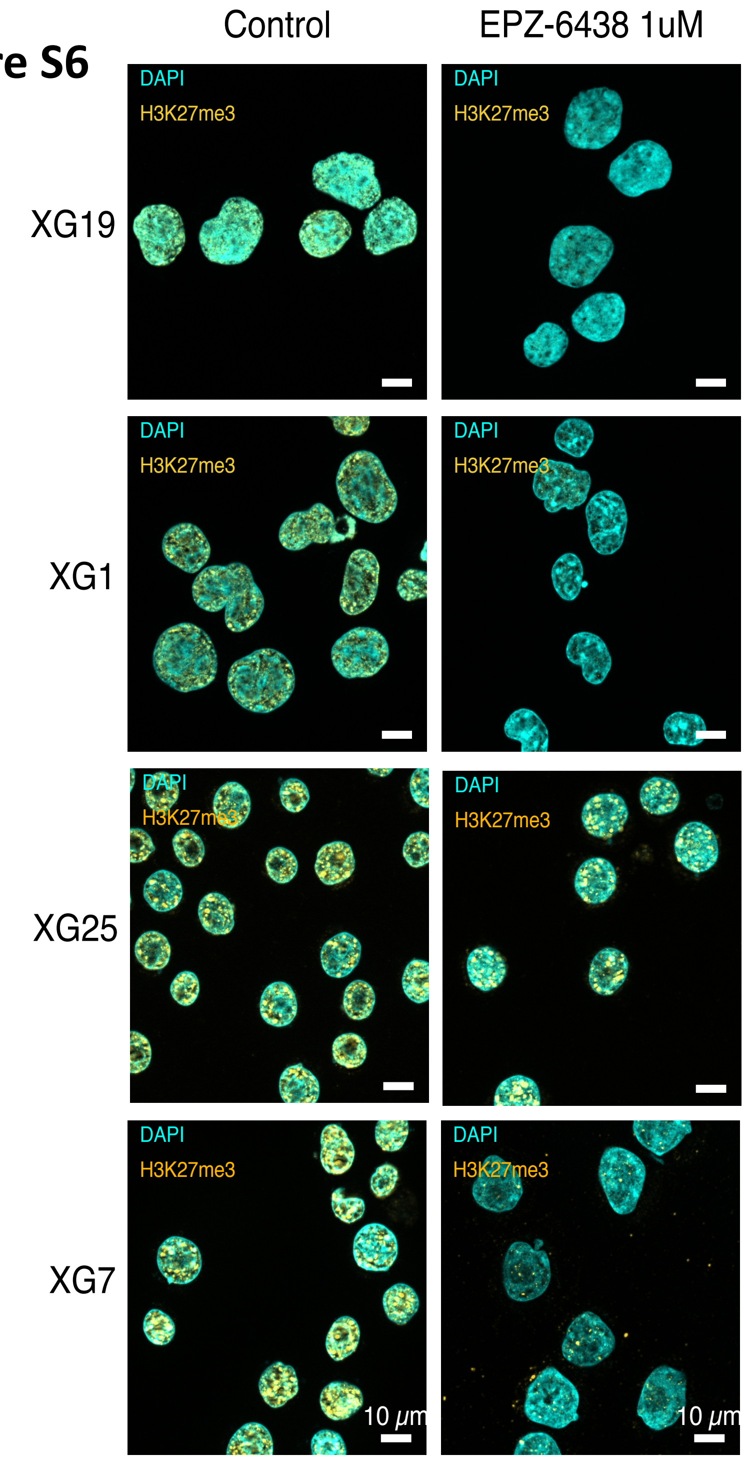

EPZ-6438 leads to H3K27me3 loss in HMCLs

The H3K27me3 status of HMCLs after EPZ-6438 treatment (370nm and 1uM) was evaluated by immunofluorescence using a rabbit anti-human primary antibody against H3K27me3 and AF-555-conjugated donkey anti-rabbit secondary antibody.

Supplementary Figure S7

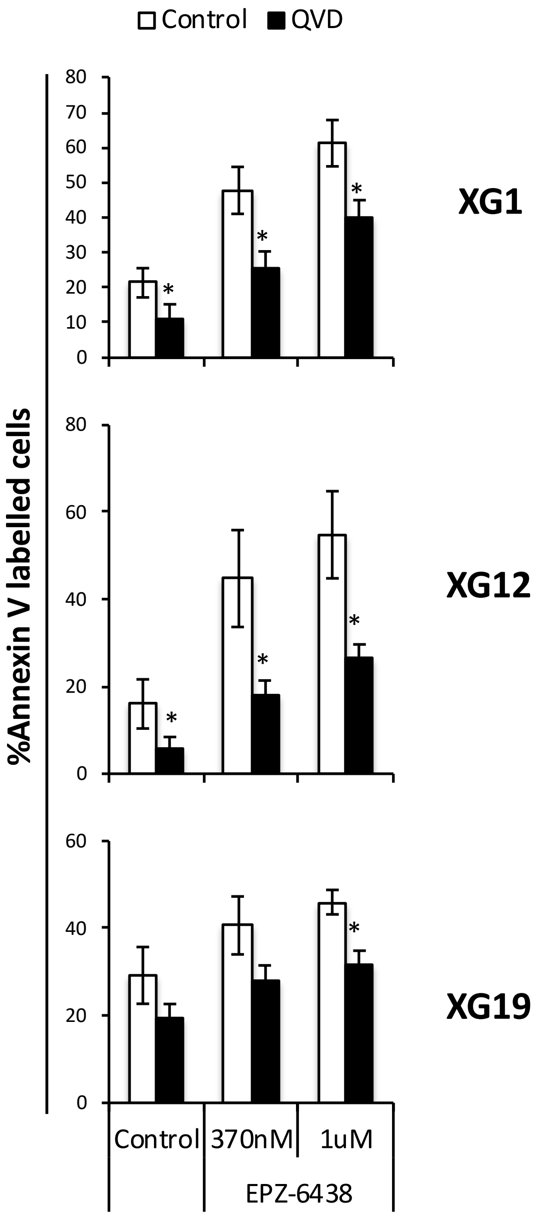

EPZ-6438-induced apoptosis is partially rescued by QVD pan caspase inhibitor. The cells were treated for 8 days with 20 µM QVD associated or not with 370nM or 1µM of EZH2 inhibitor EPZ-6438. Apoptosis induction was analyzed using Annexin V PE staining by flow cytometry. Data are mean values ± standard deviation (SD) of five experiments. * indicates a significant difference compared to control using a Wilcoxon test for pairs ($P \leq 0.05$).

Supplementary Figure S8

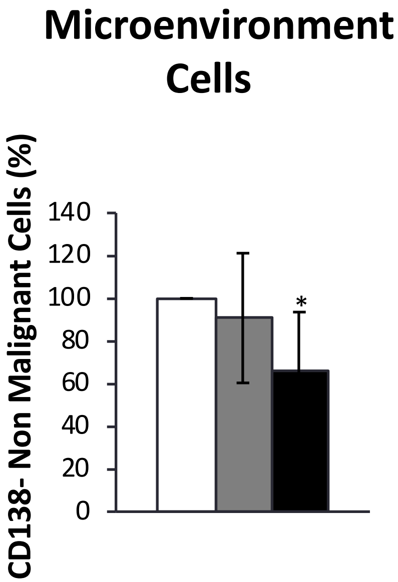

EZH2 inhibition induces mortality of primary MM cells from patients. Mononuclear cells from 17 patients with MM were treated with EZH2 inhibitor EPZ-6438. At day 8 of culture, the viability and total cell counts were assessed and the percentage of bone marrow non-myeloma cells was determined by flow cytometry. Results are median values of the numbers of myeloma cells in the culture wells. * indicates a significant difference compared to control using a Wilcoxon test for pairs ($P \leq 0.05$).

Control EPZ-6438 370nM EPZ-6438 1uM

Supplementary Figure S9

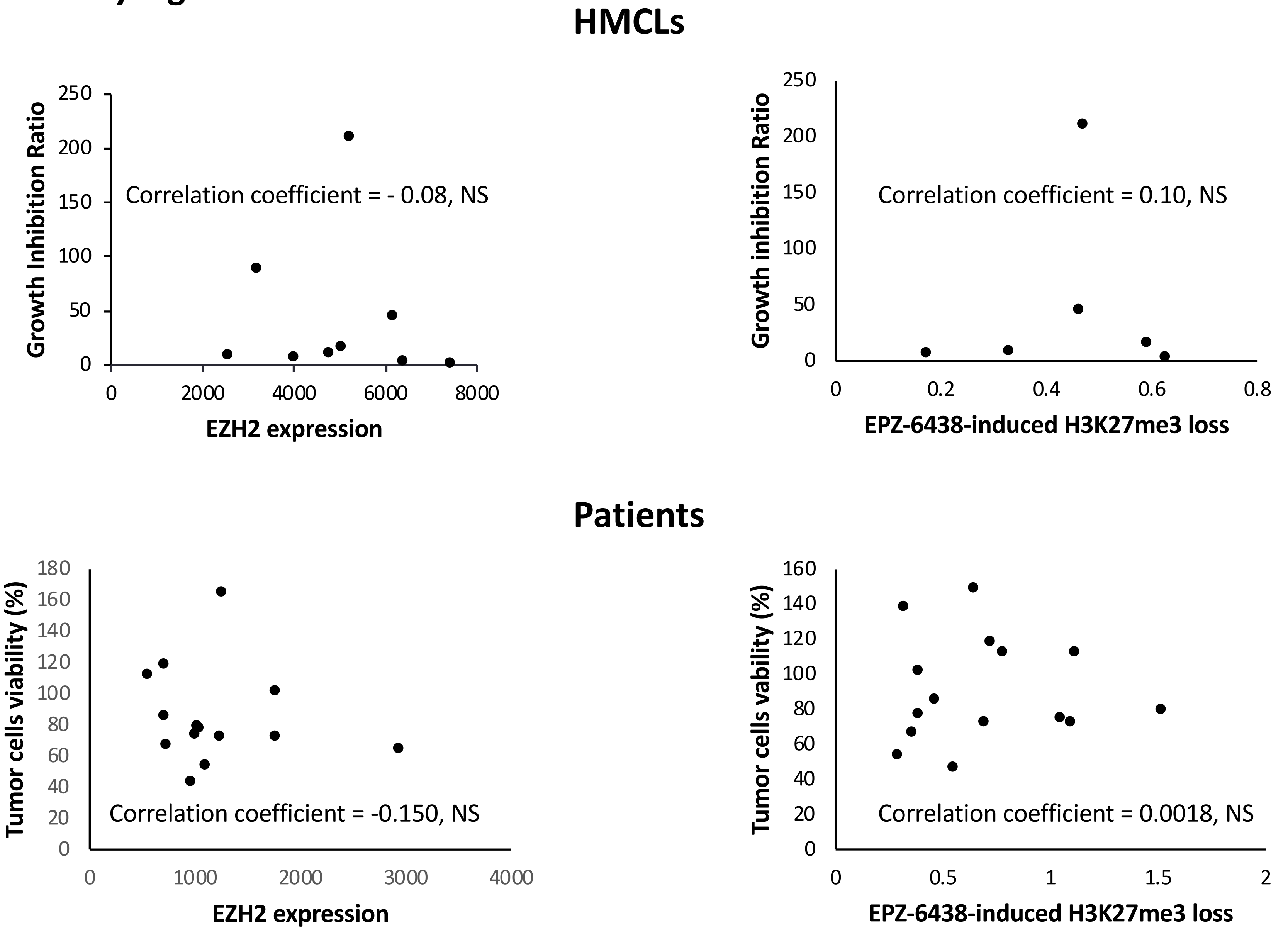

Correlation between EZH2 expression/H3K27m3 staining and drug response to EZH2 inhibitor in HMCLs and patients. HMCLs : Growth inhibition ratio at day 15 compared with EZH2 expression or EPZ-6438-induced H3K27me3 loss at day 8 of treatmenr. Patients : Tumor cells viability compared with H3K27me3 global staining index or EPZ-6438-induced H3K27me3 loss at day 8 of treatment. NS = Non significant

Supplementary Figure S10

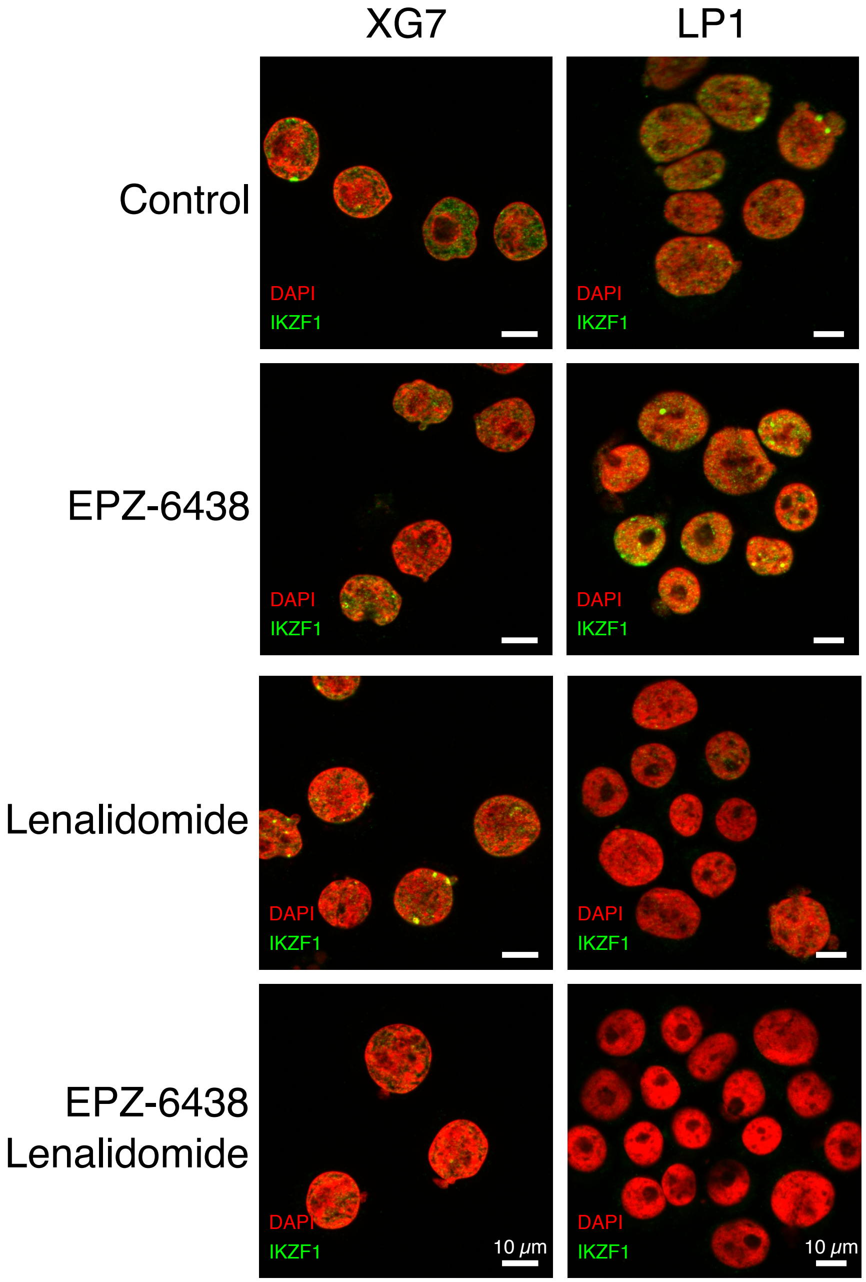

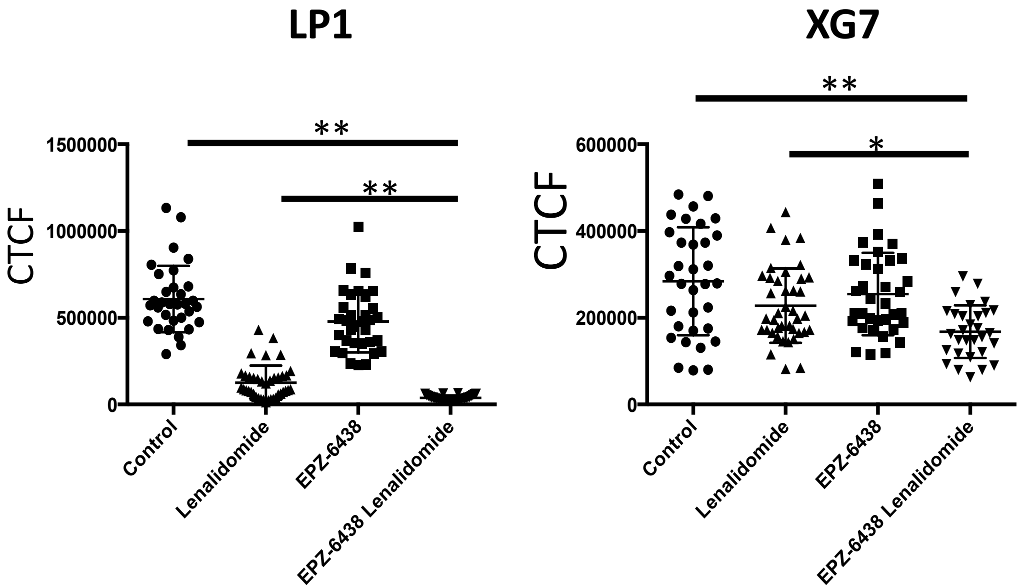

IKZF1 protein level decreases after HMCLs were treated with EPZ-6438 and Lenalidomide combination. IKZF1 expression was assessed by immunofluorescence using an anti-IKZF1 primary antibody and an AF555-conjugated secondary antibody. Corrected total cell fluorescence was measured (CTCF)

Supplementary Figure S11

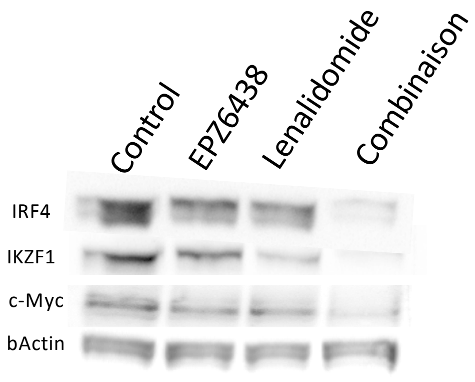

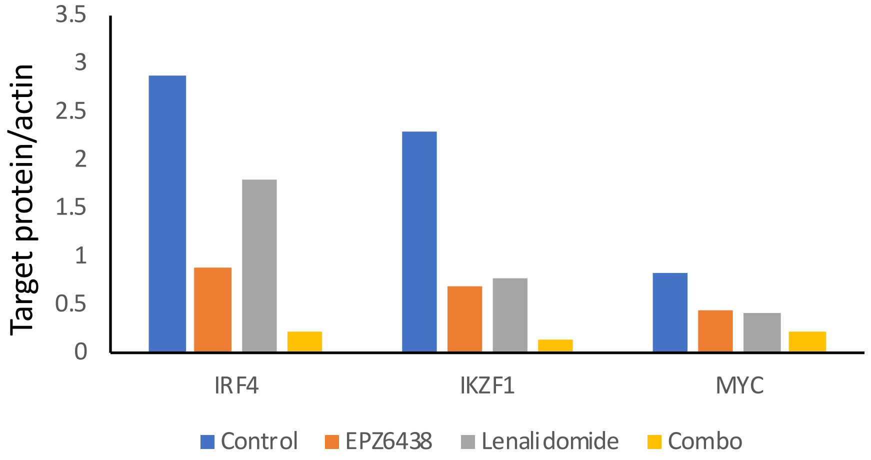

Lenalidomide targets protein levels after treatment : XG7 HMCL was treated with 2µM Lenalidomide for 2days with (Combination) or without (Lenalidomide) prior 4days-treatment with 1µM EPZ-6438. Protein levels was assessed by western blot

Supplementary Figure S12

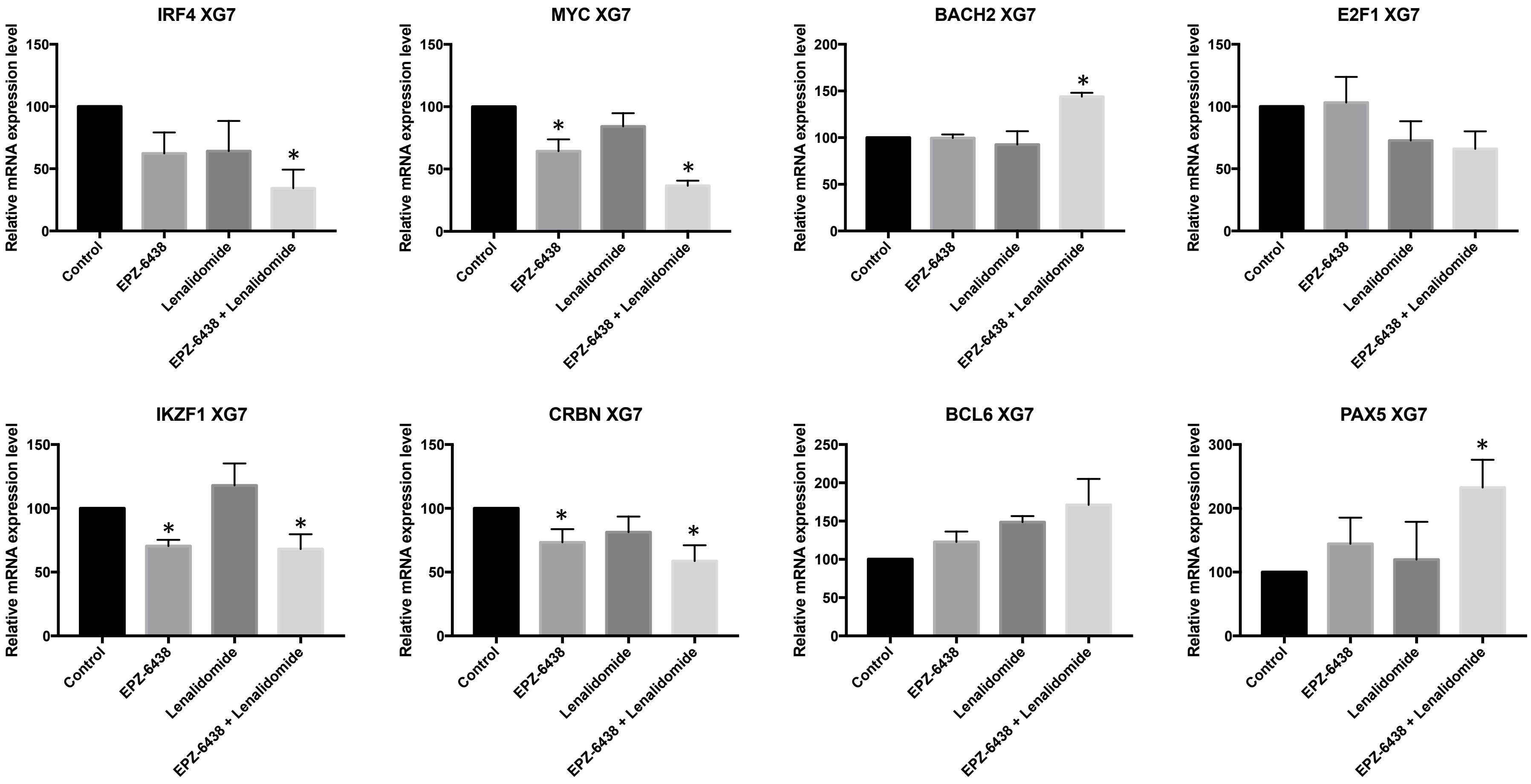

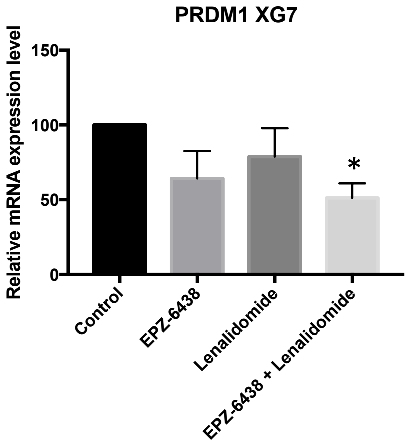

B cell transcription factors mRNA expression after treatment : XG7 HMCL was treated with 2µM Lenalidomide 2days with (Combination) or without (Lenalidomide) prior 4days-treatment with 1µM EPZ-6438. Relative mRNA expression levels were assessed by RT-qPCR.

Supplementary Figure S13

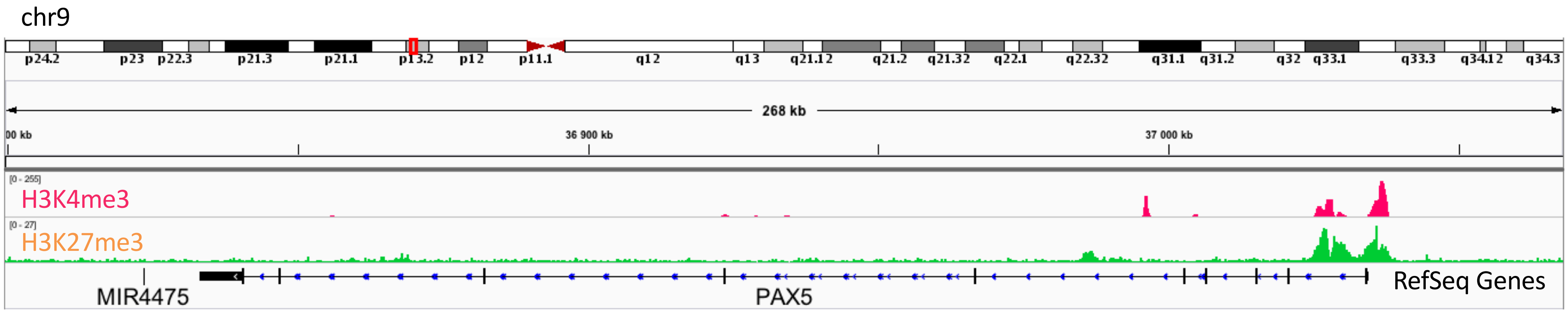

PAX5 is a bivalent gene in XG7 HMCL: H3K4me3 and H3K27me3 profile on PAX5 was assessed by ChIP-seq
